# Post-inflammatory Ileitis Induces Non-neuronal Purinergic Signaling Adjustments of Cholinergic Neurotransmission in the Myenteric Plexus

**DOI:** 10.3389/fphar.2017.00811

**Published:** 2017-11-08

**Authors:** Cátia Vieira, Fátima Ferreirinha, Maria T. Magalhães-Cardoso, Isabel Silva, Patrícia Marques, Paulo Correia-de-Sá

**Affiliations:** Laboratório de Farmacologia e Neurobiologia, Center for Drug Discovery and Innovative Medicines (MedInUP), Instituto de Ciências Biomédicas de Abel Salazar, Universidade do Porto, Porto, Portugal

**Keywords:** post-inflammatory ileitis, acetylcholine release, adenosine release, ATP release, myenteric plexus, enteric glia, interstitial cells of Cajal

## Abstract

Uncoupling between ATP overflow and extracellular adenosine formation changes purinergic signaling in post-inflammatory ileitis. Adenosine neuromodulation deficits were ascribed to feed-forward inhibition of ecto-5′-nucleotidase/CD73 by high extracellular adenine nucleotides in the inflamed ileum. Here, we hypothesized that inflammation-induced changes in cellular density may also account to unbalance the release of purines and their influence on [^3^H]acetylcholine release from longitudinal muscle-myenteric plexus preparations of the ileum of 2,4,6-trinitrobenzenesulfonic acid (TNBS)-treated rats. The population of S100β-positive glial cells increase, whereas Ano-1-positive interstitial cells of Cajal (ICCs) diminished, in the ileum 7-days after the inflammatory insult. In the absence of changes in the density of VAChT-positive cholinergic nerves detected by immunofluorescence confocal microscopy, the inflamed myenteric plexus released smaller amounts of [^3^H]acetylcholine which also became less sensitive to neuronal blockade by tetrodotoxin (1 μM). Instead, [^3^H]acetylcholine release was attenuated by sodium fluoroacetate (5 mM), carbenoxolone (10 μM) and A438079 (3 μM), which prevent activation of glial cells, pannexin-1 hemichannels and P2X7 receptors, respectively. Sodium fluoroacetate also decreased ATP overflow without significantly affecting the extracellular adenosine levels, thus indicating that surplus ATP release parallels reactive gliosis in post-inflammatory ileitis. Conversely, loss of ICCs may explain the lower amounts of adenosine detected in TNBS-treated preparations, since blockade of Ca_v_3 (T-type) channels existing in ICCs with mibefradil (3 μM) or inhibition of the equilibrative nucleoside transporter 1 with dipyridamole (0.5 μM), both decreased extracellular adenosine. Data indicate that post-inflammatory ileitis operates a shift on purinergic neuromodulation reflecting the upregulation of ATP-releasing enteric glial cells and the depletion of ICCs accounting for decreased adenosine overflow via equilibrative nucleoside transporters.

## Introduction

Inflammation of the gastrointestinal (GI) tract triggers a series of adaptive morphological, chemical and functional changes in the cellular components responsible for maintaining gut homeostasis (Sharkey and Kroese, 2001). These involve the number and chemical coding of enteric neurons, but also the relative abundance and activity of adjacent non-neuronal cells such as enteric glia, interstitial cells of Cajal, fibroblast-like cells and smooth muscle fibers, which are directly or indirectly influenced by inflammatory cells infiltrates. Adaptive cellular responses may impact on the coordination of motor function, local blood flow, GI secretions and also on the endocrine and immune reactions (Costa et al., 2000). As a matter of fact, the post-inflammatory status is frequently accompanied by significant changes in enteric motility (Pontell et al., 2009; Vieira et al., 2014).

Changes in the release of purines together with adaptive modifications of purinoceptors expression and/or function are hallmarks of inflammatory reactions in most tissues, with the GI tract being no exception (reviewed in Roberts et al., 2012). Although purinergic signaling modifications underlying inflammatory responses of the GI tract are not fully understood, the extreme plasticity of the purinergic system and its pathophysiological impact on immune reactions, enteric neuronal networking and cellular communication make drugs targeting the purinergic cascade ideal candidates for treating inflammatory GI diseases. Purines, such as ATP and adenosine, are released from activated infiltrating inflammatory cells (Marquardt et al., 1984), as well from resident neuronal and non-neuronal enteric cells (Stead et al., 1989; Bogers et al., 2000). ATP released in response to inflammatory mediators is crucial for neutrophil activation and immune defense (Lazarowski et al., 2011), but can also function as a danger signal preventing cells invasion of immune-privileged tissues, like myenteric ganglia (Bradley et al., 1997).

In healthy individuals, ATP is co-released by vesicular exocytosis from enteric neurons with other neurotransmitters, like acetylcholine (ACh; Burnstock, 1976), which is the main responsible for gut motility. Mounting evidences indicate that ATP release from non-neuronal cells is also critical to control organ functions, in both normal and stressful conditions (Bodin and Burnstock, 2001; Lazarowski et al., 2011; Mutafova-Yambolieva, 2012; Pinheiro et al., 2013; Silva et al., 2015). Non-neuronal release of ATP may be carried out by vesicular ATP transporters (VNUT) (Sawada et al., 2008; Lazarowski et al., 2011), as well as via other mechanisms involving activation of ABC proteins and hemichannels containing connexins and/or pannexins (Bodin and Burnstock, 2001; Lazarowski et al., 2011; Pinheiro et al., 2013; Carneiro et al., 2014; Timóteo et al., 2014; Silva et al., 2015).

Once released from either neuronal or non-neuronal cells, ATP modifies organ functions by activating directly ionotropic P2X and metabotropic P2Y purinoceptors or indirectly, via P1 receptors, after being metabolized into adenosine through ecto-nucleotidases. At the myenteric neuromuscular synapse, ATP transiently facilitates [^3^H]ACh release from non-stimulated nerve terminals via the activation of P2X (most probably P2X2 or P2X2/3) receptors (Duarte-Araújo et al., 2009). Fast conversion of ATP directly into AMP catalyzed by NTPDases 2 and 3 (Vieira et al., 2014) and, subsequent, formation of adenosine by ecto-5′-nucleotidase/CD73, controls evoked [^3^H]ACh release through stimulation of high-affinity excitatory A_2A_ and/or inhibitory A_1_ receptors located on nerve terminals and ganglion cells bodies of myenteric neurons, respectively (Duarte-Araújo et al., 2004a, 2009; Vieira et al., 2011). However, inflammation may lead to overexpression of NTPDase2 at the myenteric synapse (Vieira et al., 2014), which is a preferential nucleoside triphosphatase hydrolysing ADP 10 to 15 times less efficiently than ATP (Kukulski et al., 2011). This favors ADP accumulation instead of adenosine and subsequent down-regulation of enteric neuromuscular transmission through the activation of inhibitory P2Y_1_ receptors (Duarte-Araújo et al., 2009).

In contrast to ATP, adenosine is not stored nor released from synaptic vesicles. The nucleoside is involved in the fine-tuning modulation of enteric neuromuscular functions, influencing nerve-evoked neurotransmitters release, smooth muscle contractility, peristaltic reflexes and, ultimately, the GI transit (reviewed in Antonioli et al., 2011b). Membrane-bound adenosine receptor subtypes are heterogeneously distributed throughout the gut. Our group has contributed to elucidate the localization and function of all four adenosine receptor subtypes at the longitudinal muscle–myenteric plexus of the rat ileum (Vieira et al., 2011) and to uncover the complexities underlying differential activation of adenosine receptors, namely inhibitory A_1_ and excitatory A_2A_, which are major contributors to control ACh release from cholinergic enteric neurons (Duarte-Araújo et al., 2004a; Correia-de-Sá et al., 2006). Data suggest that adenosine inactivation systems, both adenosine deaminase and equilibrative nucleoside transporters, located in close proximity to the nucleoside release / production sites at the myenteric synapse are the key determinants for the predominant excitatory tone mediated by A_2A_ receptors. Under normal physiological conditions, this microenvironment restricts endogenous adenosine actions to the synaptic region where A_2A_ receptors are concentrated on cholinergic nerve terminals, thus preventing activation of other receptor subtypes located more abundantly in extrasynaptic regions (e.g., myenteric cell bodies and enteric glia). Yet, this scenario may change under pathological conditions (see e.g., De Man et al., 2003; Antonioli et al., 2011a; Zoppellaro et al., 2013).

Concerning adenosine production, our group demonstrated that the ecto-nucleotidase pathway contributes only partially to the total interstitial adenosine concentration in the rat myenteric plexus (Correia-de-Sá et al., 2006). Adenosine released as such from either neuronal or non-neuronal cells seems to be the main source of endogenous adenosine in the enteric nervous system (Duarte-Araújo et al., 2004a). This release is sought to be mediated by facilitated diffusion via equilibrative nucleoside transporters (Duarte-Araújo et al., 2004a; Correia-de-Sá et al., 2006) existing in interstitial cells of Cajal (ICCs) (Vieira et al., 2014), among other cells. Another potential source of endogenous adenosine could be adenosine 3′,5′-cyclic monophosphate (cAMP) extruded from activated cells, which can be converted to AMP and then to adenosine by ecto-phosphodiesterase and ecto-5′-nucleotidase/CD73, respectively (Giron et al., 2008). Likewise, β-NAD^+^ released from stimulated enteric nerve varicosities (Hwang et al., 2011; Durnin et al., 2013) could also serve as adenosine precursor. But again, the last two sources of adenosine depend on the activity of ecto-5′-nucleotidase/CD73, which apparently accounts only partially for endogenous adenosine accumulation in the myenteric plexus of the rat ileum (Vieira et al., 2014).

Uncoupling between ATP overflow and extracellular adenosine formation has been observed in post-inflammatory ileitis (Vieira et al., 2014). This situation disrupts the purinergic control of gut motility and has been ascribed to feed-forward inhibition of ecto-5′-nucleotidase/CD73 by high extracellular levels of adenine nucleotides together with augmentation of adenosine deaminase (ADA) activity in the inflamed ileum. While these findings explain, at least partially, the loss of adenosine neuromodulation in the inflamed ileum, they miss the point regarding enhancement of the ATP-mediated tone. In this study, we hypothesized that inflammation-induced changes in the density of specific enteric resident cells could also account to unbalance the release of purines and, thus, their influence on evoked [^3^H]ACh release from longitudinal muscle-myenteric plexus preparations of rats with TNBS-induced post-inflammatory ileitis.

## Materials and Methods

### TNBS-Induced Intestinal Inflammation Rat Model

The animals were provided free access to standard laboratory chow and water. Animal care and experimental procedures were carried out in accordance with the United Kingdom Animals (Scientific Procedures) Act 1986 and followed the European Communities Council Directive of 24 November 1986 (86/609/EEC) and the National Institutes of Health Guide for Care and Use of Laboratory animals (NIH Publications No. 80-23) revised 1996. All studies involving animals are reported in accordance with ARRIVE guidelines for reporting experiments involving animals (McGrath et al., 2010). This study and all its procedures were approved by the Ethics Committee and the Animal Welfare Responsible Organism of ICBAS-UP (Decision N° 224/2017).

Rats (Wistar, ∼200 g; CharlesRiver, Barcelona, Spain) of both gender were kept at a constant temperature (21°) and a regular light- (06.30–19.30 h) dark (19.30–06.30 h) cycle, with food and water) and a regular light- (06.30–19.30 h) dark (19.30–06.30 h) cycle, with food and water *ad libitum*. Intestinal inflammation was produced by the instillation of 2,4,6-trinitrobenzenesulfonic acid (TNBS) into the lumen of the rat ileum, according to the procedures described in a previous study from our group (Vieira et al., 2014) and confirmed in haematoxylin-eosin stained histological sections using the Pontell and Jergens criteria (Jergens, 1999; Wirtz and Neurath, 2007; Engel et al., 2008; Pontell et al., 2009). After a fasting period of 4–8 h with free access to drinking water, rats underwent median laparotomy under anesthesia with medetomidine (10 mg/Kg) plus ketamine (75 mg/Kg) subcutaneously. At the end of the procedure animals were retrieved with atipamezole (10 mg/Kg) subcutaneously. The terminal ileal loop was gently exteriorized, and TNBS (40 mM, 1 mL) was injected through the enteric wall into the lumen of the ileum with a 29G (0.3366 mm OD) beveled needle, 10 cm proximal to the ileocolonic junction. Controls received 1 mL of 0.9% saline. Sixty minutes after surgery, the rats were allowed to eat and drink *ad libitum*. After surgery, pain was controlled with tramadol hydrochloride (10 mg/Kg) subcutaneously. To have a control of the time course of body weight loss and recovery after injection of TNBS, rats were weighed prior to TNBS administration and daily following surgery. Animals with intestinal inflammation (TNBS) transiently lose weight for 3–4 days after surgery and regain weight thereafter. Seven days after surgery, animals were sacrificed following an overnight fasting period.

### [^3^H]Acetylcholine Release Experiments

Eight centimeters sections of the rat ileum not including the terminal one centimeter and the injected proximal portion were used. The longitudinal muscle strip with the myenteric plexus attached was separated from the underlying circular muscle according to the method of Paton and Vizi (1969). This preparation is abundant in cholinergic neurons, mainly excitatory neurons projecting to the longitudinal muscle (25%) that receive inputs from intrinsic primary afferents (26%) and from ascending and descending pathways (17%) (Costa et al., 1996).

The procedures used for labeling the preparations and measuring evoked [^3^H] acetylcholine ([^3^H]ACh) release were previously described (Vizi et al., 1984; Duarte-Araújo et al., 2004a; Correia-de-Sá et al., 2006; Vieira et al., 2011) and used with minor modifications. Isolated longitudinal muscle-myenteric plexus (LM-MP) strips were subdivided into 2-cm pieces, which were randomly mounted in 365 μL chambers of a semi-automated 12-sample superfusion system (SF-12 Suprafusion 1000, Brandel, Gaithersburg, MD, United States) heated at 37°C. The preparations were superfused with gassed (95% O_2_ and 5% CO_2_) Tyrode’s solution containing (mM): NaCl 137, KCl 2.7, CaCl_2_ 1.8, MgCl_2_ 1, NaH_2_PO_4_ 0.4, NaHCO_3_ 11.9 and glucose 11.2. After a 30-min equilibration period, nerve terminals were labeled during 40 min with 1 μM of [^3^H]choline (specific activity 5 μCi/nmol) under electrical field stimulation (1 Hz frequency, 1 ms pulse width, 75 mA) using two platinum-made grid electrodes placed above and below the muscle strip (transmural EFS stimulation). Following loading, the washout superfusion (1 ml/min) of the preparations was performed during 80 min with Tyrode’s solution supplemented with the choline uptake inhibitor, hemicholinium-3 (10 μM). Tritium outflow was evaluated by liquid scintillation spectrometry (TriCarb2900TR, Perkin Elmer, and Boston, MA, United States; % counting efficiency: 56 ± 2%) in 0.6 ml bath samples automatically collected every 1 min using the SF-12 suprafusion system. [^3^H]ACh release was evoked by two periods of EFS (S_1_ and S_2_), each consisting of 200 square wave pulses of 1 ms duration delivered at a 5-Hz frequency. The area of the peak corresponding to evoked [^3^H]ACh release was calculated as the sum of the differences between the total radioactivity present in the 4 samples collected after stimulus application and the basal tritium outflow (see **Figure 3A**). Baseline values were inferred by linear regression of the radioactivity decay immediately before stimulus and after its return to baseline (e.g., Duarte-Araújo et al., 2004a; Correia-de-Sá et al., 2006; Vieira et al., 2011, 2014). Test drugs were added 8 min before S_2_ and were present up to the end of the experiments. The change in the ratio between the evoked [^3^H]ACh release during the two stimulation periods (S_2_/S_1_) relative to that observed in control conditions (in the absence of test drugs) was taken as a measure of the effect of the tested drugs; in the absence of test drugs, the calculated S_2_/S_1_ ratios were 0.83 ± 0.07 (*n* = 6) and 0.80 ± 0.09 (*n* = 6) in control and TNBS-treated samples, respectively (see **Figure 5A**). Positive and negative values represent facilitation and inhibition of evoked [^3^H]ACh release, respectively. None of the drugs significantly (*P* > 0.05) changed the basal tritium outflow.

### Release of ATP and Adenine Nucleosides (Adenosine plus Inosine)

The procedures used to measure ATP and adenine nucleosides were previously described (Vieira et al., 2014). Experiments were performed using an automated perfusion system for sample collecting for given time periods, therefore improving the efficacy of HPLC (with diode array detection) and bioluminescence analysis. After a 30-min equilibration period, the preparations were incubated with 1.8 mL gassed Tyrode’s solution, which was automatically changed every 15 min by emptying and refilling the organ bath with the solution in use. The preparations were electrically stimulated once, 15 min after starting sample collection (zero time), using 3000 square wave pulses of 1-ms duration delivered at a 5-Hz frequency. In these experiments, only the sample collected before stimulus application and the two samples collected after stimulation were retained for analysis. Bath aliquots (50–250 μL) were frozen in liquid nitrogen immediately after collection, stored at -20°C (the enzymes are stable for at least 4 weeks) and analyzed within 1 week of collection by HPLC with diode array detection (Finigan Thermo Fisher Scientific System LC/DAD, equipped with an Accela Pump coupled to an Accela Autosample, a diode array detector and an Accela PDA running the X-Calibur software chromatography manager). Chromatographic separation was carried out through a Hypersil GOLD C18 column (5 μM, 2.1 mm × 150 mm) equipped with a guard column (5 μm, 2.1 mm × 1 mm) using an elution gradient composed of ammonium acetate (5 mM, with a pH of 6 adjusted with acetic acid) and methanol. During the procedure the flow rate was set at 200 μL per min and the column temperature was maintained at 20°C. The autosampler was set at 4°C and 50 μL of standard or sample solution was injected, in duplicate, for each HPLC analysis. In order to obtain chromatograms and quantitative analysis with maximal sensibility, the diode array detection wavelength was set at 259 nm for adenosine and 248 nm for inosine (**Supplementary Figure S1**).

The ATP content of the same samples was evaluated in parallel with the luciferin-luciferase ATP bioluminescence assay kit HS II (Roche Applied Science, Indianapolis, IN, United States). Luminescence was determined using a multi detection microplate reader (SynergyHT, BioTek Instruments).

Stimulation-evoked release of adenine nucleotides and nucleosides was calculated by subtracting the basal release, measured in the sample collected before stimulation, from the total release of adenine nucleotides and nucleosides determined in the sample collected immediately after stimulus application.

### Myographic Recordings of Ileal Contractile Activity

The contractile activity of the LM-MP was recorded as previously described (Vieira et al., 2009, 2011; Mendes et al., 2015). Ileum strips from control and TNBS-treated rats were mounted along the longitudinal axis in 14 ml capacity perfusion chambers connected to isometric force transducers. The changes in tension were recorded continuously with a PowerLab data acquisition system (Chart 5, v.4.2; AD Instruments, United States). Tissues were preloaded with 0.5 g of tension and allowed to equilibrate for 90 min under continuous superfusion with gassed (95% O_2_ and 5% CO_2_) Tyrode’s solution at 37°C.

### Immunofluorescence Staining and Confocal Microscopy Observation

LM-MP fragments were isolated from the rat ileum as previously described. The LM-MP fragments were stretched to all directions and pinned onto Petri dishes coated with Sylgard^®^. The tissues, then, were fixed in PLP solution (paraformaldehyde 2%, lysine 0.075 M, sodium phosphate 0.037 M, sodium periodate 0.01 M) for 16 h at 4°C, unless stated otherwise. Following fixation, the preparations were washed three times for 10 min each using 0.1 M phosphate buffer. At the end of the washout period, tissues were cryoprotected during 16 h with a solution containing anhydrous glycerol 20% and phosphate buffer 0.1 M at 4°C and, then, stored at -20°C for further processing. Once defrosted, tissue fragments were washed with phosphate saline buffer (PBS) and incubated with a blocking buffer, consisting in fetal bovine serum 10%, bovine serum albumin 1%, triton X-100 1% in PBS, for 2 h; washout was facilitated by constant stirring of the samples. After blocking and permeabilization, samples were incubated with selected primary antibodies (see **Table 1**) diluted in the incubation buffer (fetal bovine serum 5%, serum albumin 1%, Triton X-100 1% in PBS), at 4°C, for 48 h. For double immunostaining, antibodies were combined before application to tissue samples. Please note that ICCs immunofluorescence staining using antibodies against Ano-1 and c-Kit required the use of acetone as tissue fixative, which difficult double immunostaining with other primary antibodies. For the c-Kit/M_3_ double immunostaining, tissues were fixed with acetone/formaldehyde solution (50% acetone, 2% PFA in PBS) for 10 min at 4°C, whereas the best results with Ano-1 antibody were obtained in tissues fixed with 100% acetone for 10 min at -20°C. Following the washout of primary antibodies with PBS supplemented with Triton X 1% (3 cycles of 10 min), tissue samples were incubated with species-specific secondary antibodies in the dark for 2 h, at room temperature. In some experiments tetramethylrhodamine-conjugated α-bungarotoxin (BTX-rhod, 1.25 μM) was incubated together with secondary antibodies to label ionotropic nicotinic receptors containing α7 subunits. Finally, tissue samples were mounted on optical-quality glass slides using VectaShield as mounting media (VectorLabs) and stored at 4°C. Observations were performed and analyzed with a laser scanning confocal microscope (Olympus FV1000, Tokyo, Japan).

**Table 1:** Primary and secondary antibodies used in immunohistochemistry experiments.

Antigen	Code	Species	Dilution	Supplier
**Primary antibodies**				
NF200	ab8135	Rabbit (rb)	1:1000	ABCAM
nNOS	ab1376	Goat (gt)	1:300	ABCAM
S100β	Ab868	Rabbit (rb)	1:400	ABCAM
Ano-1	Ab53212	Rabbit (rb)	1:100	ABCAM
P2X7	APR-004	Rabbit (rb)	1:50	Alomone
M3	AMR-006	Rabbit (rb)	1:50	Alomone
ENT1	ANT-051	Rabbit (rb)	1:100	Alomone
VaChT	AB1588	Guinea-pig (gp)	1:500	Chemicon
ChAT	AB 144P	Goat (gt)	1:100	Chemicon
GFAP	MAB360	Mouse (ms)	1:600	Chemicon
Vimentin	M0725	Mouse (ms)	1:150	Dako
c-Kit	SC-1494	Goat (gt)	1:50	Santa Cruz
CD206	SC-34577	Goat (gt)	1:50	Santa Cruz
CD11B/αM	Sc-53086	Mouse (ms)	1:50	Santa Cruz
PGP 9.5	7863-1004	Mouse (ms)	1:750	Serotec
**Secondary antibodies**				
Alexa Fluor 488 anti-rb	A-21206	Donkey	1:1000	Molecular probes
Alexa Fluor 488 anti-ms	A21202	Donkey	1:1000	Molecular probes
Alexa Fluor 568 anti-gt	A11057	Donkey	1:1000	Molecular probes
Alexa Fluor 568 anti-ms	A-10037	Donkey	1:1000	Molecular probes
Alexa Fluor 633 anti-ms	A21052	Goat	1:1000	Molecular probes
TRITC 568 anti-gp	706-025-148	Donkey	1:150	Jackson Immuno Res.
Dylight 649 anti-gp	706-025-148	Donkey	1:100	Jackson Immuno Res.


The images were stored in TIFF format with the same resolution and, subsequently, analyzed with the ImageJ^®^ software version 1.46r (National Institutes of Health) in order to quantify the density of stained cell constituents of the LM-MP. Settings such as the area, the integrated density and the mean gray value were measured systematically in all analyzed images; background settings were obtained from an area of the section untreated with the primary-antibody. The values obtained were used to calculate the corrected total cryosection fluorescence (CTCF) using a formula published in the website^1^.

Co-localization was assessed by calculating the Pearson’s linear correlation coefficient (ρ) and the staining overlap for each confocal micrograph stained with two fluorescent dyes using the Olympus Fluoview 4.2 Software (Olympus FV1000, Tokyo, Japan) (see e.g., Barros-Barbosa et al., 2015, 2016a,b). The ρ value is a measure of pixel-by-pixel covariance in the signal levels of two images (stainings) and varies between +1 and -1, inclusive, where 1 is total positive correlation, 0 is no correlation, and -1 is total negative correlation; because it subtracts the mean intensity from each pixel’s intensity value, the ρ coefficient is independent of signal levels and signal offset (background). Thus, the Pearson’s linear correlation coefficient can be measured in two-color images without any form of preprocessing, making it both simple and relatively safe from user bias (Dunn et al., 2011). Because (1) ρ may be less sensitive to differences in signal intensity between the components of an image caused by different labeling with fluorocromes, photobleaching or different settings of amplifiers, and (2) the negative values of ρ are difficult to interpret when the degree of overlap is the quantity to be measured, the subtraction of the averages of the two colors can be omitted to create the overlap coefficient, which varies between +1 (total overlap) and 0 (no overlap); as with the Pearson’s, this coefficient is not dependent on the magnitude of the signal (gain), but does depend on the background.

### Materials and Solutions

2,4,6-trinitrobenzenesulphonic acid (TNBS); carbenoxolone, choline chloride, paraformaldehyde (prills), lysine, sodium periodate, anhydrous glycerol, fetal bovine serum (Sigma, St Louis, MO, United States); serum albumin, triton X-100, methanol, potassium dihydrogen phosphate (KH_2_PO_4_) (Merck, Darmstadt, Germany); 3-[[5-(2,3-dichlorophenyl)-1H-tetrazol-1-yl]methyl]pyridine hydrochloride (A438079), mibefradil dihydrochloride, N^G^-nitro-L-arginine methyl ester hydrochloride (L-NAME), 2′,3′-O-(2,4,6-trinitrophenyl)adenosine-5′-triphosphate tetra(triethylammonium) salt (TNP-ATP); tetrodotoxin (TTX) (Tocris Cookson Inc., United Kingdom); Sodium Fluoroacetate (Supelco); Apamin was from Abcam Biochemicals (Cambridge, United Kingdom); tetramethylrhodamine-conjugated α-bungarotoxin (BTX-rhod) was from ThermoFisher Scientific (Waltham, MA, United States); [methyl-^3^H]-choline chloride (ethanol solution, 80 Ci mmol^-1^) (Amersham, United Kingdom); ATP bioluminescence assay kit HS II (Roche Applied Science, Indianapolis, IN, United States); medetomidine hydrochloride (Domitor, Pfizer Animal Health); atipamezole hydrochloride (Antisedan, Orion, Espoo, Finland); ketamine hydrochloride (Imalgene, Merial, Lyon, France); Sodium chloride 0.9%, tramadol hydrochloride (Labesfal, Santiago de Besteiros, Portugal).

All drugs were prepared in distilled water. All stock solutions were stored as frozen aliquots at -20°C. Dilutions of these stock solutions were made daily and appropriate solvent controls were done. No statistically significant differences between control experiments, made in the absence or in the presence of the solvents at the maximal concentrations used (0.5% v/v), were observed. The pH of the perfusion solution did not change by the addition of the drugs in the maximum concentrations applied to the preparations.

### Presentation of Data and Statistical Analysis

The values are expressed as mean ± SEM, with n indicating the number of animals used for a particular set of experiments. Statistical analysis of data was carried out using unpaired Student’s *t*-test with Welch correction. *P* < 0.05 represents significant differences.

## Results

### Post-inflammatory Ileitis Causes an Increase in Enteric Glial Cells (Types III and IV) and a Partial Loss of Pacemaker Interstitial Cells of Cajal (ICCs)

**FIGURE 1:**
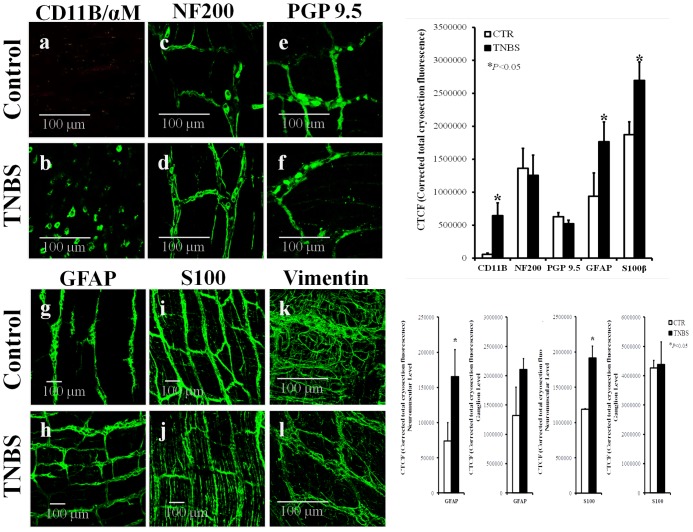
Confocal micrographs of whole-mount preparations of the longitudinal muscle-myenteric plexus of the ileum of control (CTR) and TNBS-treated rats. Z-stacks illustrate the immunoreactivity against CD11B/αM (OX42) (marker of inflammatory cells) **(a,b)**, NF200 (neurofilament expressed in neurons) **(c,d)**, PGP9.5 (pan-neuronal cell marker) **(e,f)**, GFAP **(g,h)** and S100β **(i,j)** (enteric glial cells markers), and vimentin (intermediate filament of mesenchymal cells, like ICCs and FLCs) **(k,l)**. Images are representative of at least four different animals per group, except for vimentin where only two rats were analyzed in each group. Scale bars = 100 μm. Bar charts at the right hand-side panels represents mean ± SEM of corrected total cryosection fluorescence (CTCF) staining for each cell marker; CTCF staining discriminated by ganglion level and neuromuscular region are also shown for GFAP and S100β antibodies. ^∗^*P* < 0.05 (unpaired Student’s *t*-test with Welch correction) represent significant differences from control animals.

Structural changes accompanied by neuronal cell death have been observed in chronic intestinal inflammation (Sanovic et al., 1999; Linden et al., 2005; Venkataramana et al., 2015). However, we found no obvious changes in the amount of neurons stained positively against (1) neurofilament NF200 expressed predominantly in Dogiel type I and II neurons (Hu et al., 2002), and (2) a pan-neuronal marker, protein gene product 9.5 (PGP 9.5), in the myenteric plexus 7 days after instillation of TNBS into the lumen of ileum compared to control rats treated with saline (**Figures 1c-f**). This is compatible with Moreels et al. (2001) findings showing that TNBS-induced ileitis in the rat lacks the chronic inflammatory phase and is characterized by an (sub)acute transmural inflammation that is accompanied by functional abnormalities of neuronal activity, which persists for at least 8 weeks without obvious neuronal loss (Stewart et al., 2003; Nurgali et al., 2007). Notwithstanding this, these authors found alterations in longitudinal muscle contractility which was attributed to structural thickness of the ileal wall.

Confocal micrographs show that neuronal cells are grouped in small clusters (enteric ganglia), which are interconnected by nerve fiber bundles from whom emerge small diameter nerve terminals (see e.g., Furness, 2006). Inflammatory infiltrates consisting of monocytes/macrophages exhibiting immunoreactivity against the integrin CD11B/OX42 were found surrounding the myenteric plexus of TNBS-treated rats (**Figure 1b**), but not in preparations from control animals (**Figure 1a**). The absence of inflammatory cells inside myenteric ganglia has been observed before (see also **Figures 3C**, **6**) and this is why myenteric ganglia are considered an immune-privileged tissue (Bradley et al., 1997).

Enteric glial cells express astrocytic cell markers, including the intermediate filament glial fibrillary acidic protein (GFAP) and the calcium-binding protein S100β (Gulbransen and Sharkey, 2012). Subtypes of enteric glia are classified in: type I, intraganglionic cells; type II, within interganglionic fibers; type III, form a matrix in the extraganglionic region remaining in close association with neuronal bundles; and type IV, elongated glia running with nerve fibers within the musculature (Boesmans et al., 2015). **Figures 1g–j** (and adjacent bar graphs) show that the myenteric plexus of rats treated with TNBS exhibits increased amounts of GFAP- and S100β-positive glial cells. Major differences in GFAP- and S100β-immunostaining were found at the neuromuscular region, meaning that TNBS treatment affects predominantly glial cells in the extraganglionic (glial type III) and intramuscular (glial type IV) regions. Thus, in contrast to the absence of significant structural changes in the neuronal cell population, TNBS-induced ileitis stimulates enteric glial cells proliferation (Bradley et al., 1997) or, at least, the increase in expression and/or synthesis of glial protein cell markers (Cabarrocas et al., 2003), in a similar manner to that observed in astrocytes of the CNS (Lhermitte et al., 1980; Rühl et al., 2004).

**FIGURE 2:**
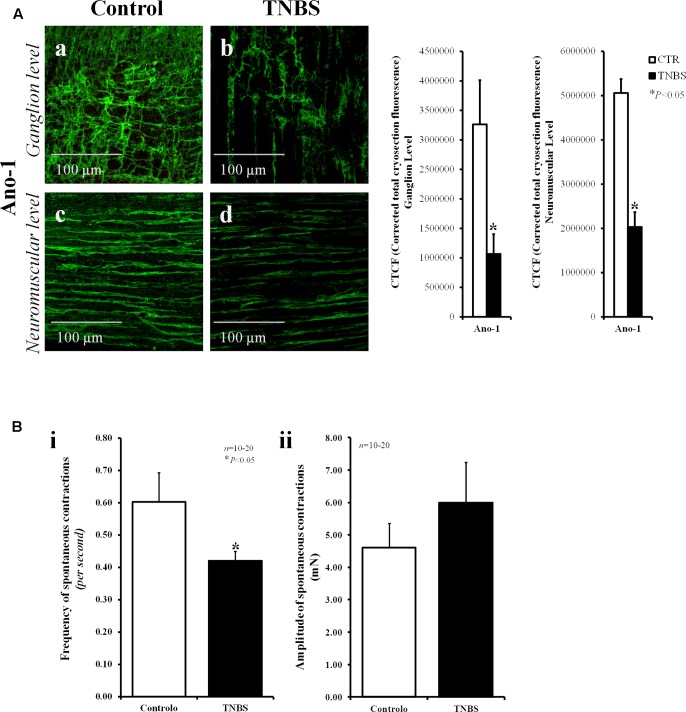
The partial loss of anoctamine-1 (Ano-1)-positive ICCs in the myenteric plexus of TNBS-treated rats correlates with the reduction in the frequency of spontaneous contractions of the rat ileum. **(A)** Confocal micrographs of whole-mount preparations of the ileum of control and TNBS-injected rats taken at the myenteric ganglion level **(a,b)** and at the longitudinal smooth muscle layer **(c,d)**. Shown is the immunoreactivity against Ano-1, a calcium-activated chloride channel involved in the pacemaker activity of ICCs. Images are representative of at least five different animals per group. Scale bars = 100 μm. Bar charts at the right hand-side panel represents mean ± SEM of corrected total cryosection fluorescence (CTCF) of Ano-1 staining at the ganglion level and at the neuromuscular region. **(B)** Histograms representing the frequency **(i)** and the amplitude **(ii)** of spontaneous myographic contractions of the ileum from control rats and TNBS-treated animals. The data are mean ± SEM of an *n* number of animals. ^∗^*P* < 0.05 (unpaired Student’s *t*-test with Welch correction) represents significant differences from control animals.

Interstitial cells of Cajal (ICCs) are known to act as pacemaker cells and integrators of nerve activity and smooth muscle contraction (Sanders, 1996). The number of these cells can significantly change in pathological conditions (Ekblad et al., 1998). Using vimentin, a characteristic intermediate filament of mesenchymal cells like ICC/FLC, we show here that the density of vimentin-positive cells decrease significantly in the myenteric plexus of TNBS-induced ileitis (**Figure 1l**) compared to control preparations (**Figure 1k**); the bar chart does not show data regarding the corrected total cryosection fluorescence (CTCF) staining of vimentin in control and TNBS-treated rats because only two animals per group were analyzed. Notwithstanding this, our data show that staining with anoctamine-1 (Ano-1), a calcium-activated chloride channel involved in the pacemaker activity of ICCs (Gomez-Pinilla et al., 2009), also decreased in density 7-days after instillation of TNBS into the lumen of the rat ileum (**Figure 2A**). Bar charts next to the confocal micrographs show that the partial loss of Ano-1 immunoreactivity was detected focusing both in the ganglion layer (myenteric stellate cells; **Figures 2Aa,b**) and in the neuromuscular region (intramuscular spindle-shape cells, **Figures 2Ac,d**) of inflamed LM-MP preparations.

The loss of ICC/FLC may affect the frequency of spontaneous enteric contractions (Kinoshita et al., 2007). In fact, myographic recordings demonstrate that spontaneous contractions of LM-MP preparations of the ileum of TNBS-treated animals were less frequent (*P* < 0.05) than those observed in control animals (**Figure 2Bi**). The amplitude of spontaneous contractions had a tendency to increase in TNBS-treated preparations, though without reaching statistical significance (*P* > 0.05) (**Figure 2Bii**).

### Myenteric Neurons from the Ileum of TNBS-Treated Rats Release Smaller Amounts of [^3^H]ACh, But No Relationship Exists between Cholinergic Hypoactivity and Nitrergic Inhibitory Signals

**FIGURE 3:**
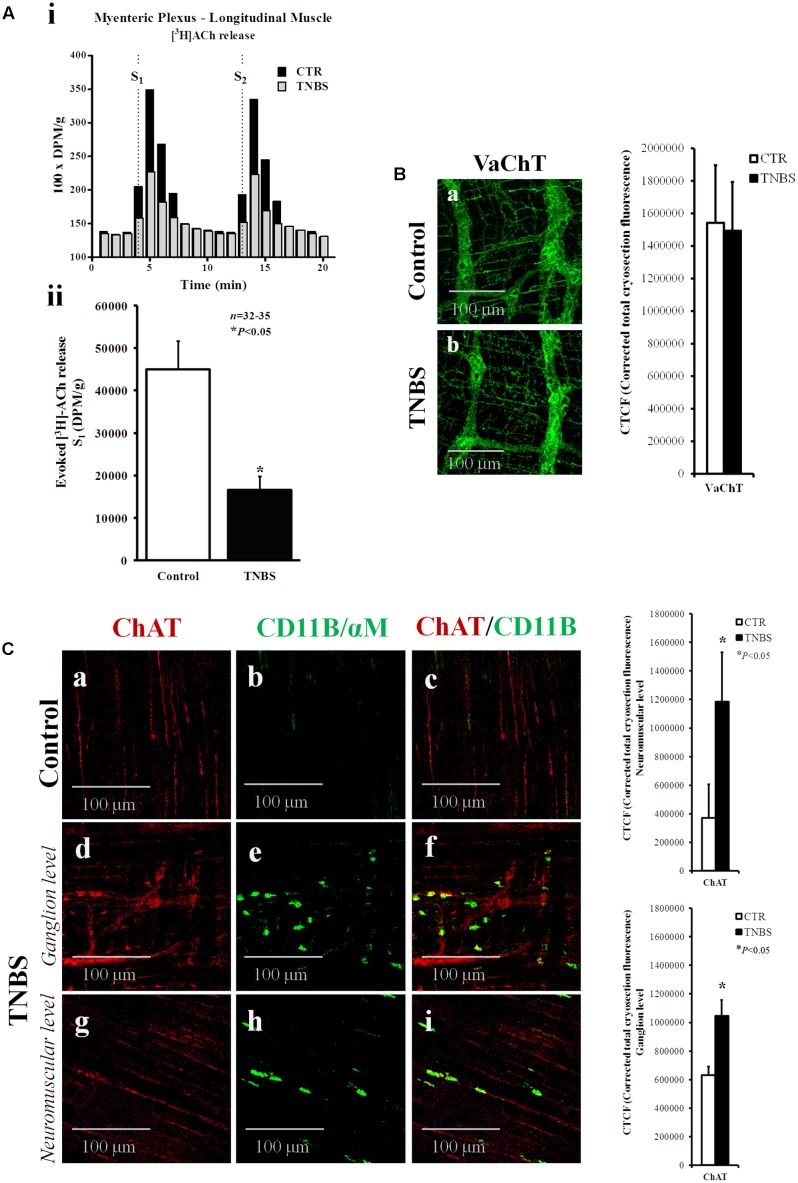
Electrically stimulated myenteric neurons of TNBS-treated rats release smaller amounts of [^3^H]ACh. **(Ai)** Ordinates represent tritium outflow from typical experiments using myenteric plexus-longitudinal muscle preparations from the ileum of control (CTR) and TNBS-treated rats expressed in disintegrations per min (DPM)/g of wet tissue. Abscissa indicates the times at which samples were collected. [^3^H]ACh release was elicited by electrical field stimulation (5 Hz, 1 ms, 200 pulses) twice, starting at 4th (S_1_) and 13th (S_2_) minutes after the end of washout (zero time). **(Aii)** Shown is the amount of [^3^H]ACh released from electrically stimulated myenteric neurons of the ileum of control and TNBS-treated rats during S_1_ in DPM/g of wet tissue. ^∗^*P* < 0.05 (unpaired Student’s *t*-test with Welch correction) represents a significant difference from control animals. **(B,C)** Confocal micrographs of whole-mount preparations of the longitudinal muscle-myenteric plexus of the ileum from control and TNBS-treated rats stained against the vesicular ACh transporter (VAChT) and choline acetyltransferase (ChAT). Please note the presence of ChAT immunoreactivity inside CD11B-positive inflammatory cells surrounding myenteric ganglia and infiltrating the intramuscular layer of TNBS-treated rats. Images are representative of at least five animals per group. Scale bar = 100 μm. Bar charts at right hand-side panels represent mean ± SEM of corrected total cryosection fluorescence (CTCF) staining against VAChT and ChAT, respectively. ^∗^*P* < 0.05 (unpaired Student’s *t*-test with Welch correction) represents a significant difference from control animals.

ACh is the prime regulator of intestinal motility and is the most important excitatory neurotransmitter in the myenteric plexus (Costa et al., 1996). **Figure 3A** shows that cholinergic neurons from the myenteric plexus of TNBS-treated rats release significantly less amounts of [^3^H]ACh (see also Collins et al., 1992a; Davis et al., 1998) despite no obvious changes were detected by confocal microscopy in the density of cholinergic nerve fibers stained specifically against the vesicular acetylcholine transporter (VAChT) (Arvidsson et al., 1997) (**Figure 3B**).

Conversely, we found that TNBS-treated rats had higher immunoreactivity against choline acetyltransferase (ChAT), the enzyme that catalyzes the transfer of an acetyl group from the coenzyme, acetyl-CoA, to choline yielding ACh (**Figure 3C**). Bar charts next to the micrographs show that changes are detected both in the ganglion layer and in the neuromuscular region of TNBS-treated preparations. Increases in ChAT-immunoreactivity may be due to the presence of this enzyme inside CD11B-positive inflammatory cells surrounding myenteric ganglia and infiltrating the intramuscular layer of TNBS-treated rats (**Figure 3C**). In inflamed preparations, CD11B co-localizes with ChAT in a subset of ChAT-positive myenteric cells as indicated by the staining overlap (0.185 ± 0.091, *n* = 8, *P* < 0.001) and the Pearson’s coefficient (0.226 ± 0.082, *n* = 8, *P* < 0.001) scores. Co-localization of CD11b and ChAT indicate that inflammatory cells are able to synthesize ACh and might contribute to non-neuronal ACh release (volume cholinergic transmission) under certain conditions, like an inflammatory insult.

**FIGURE 4:**
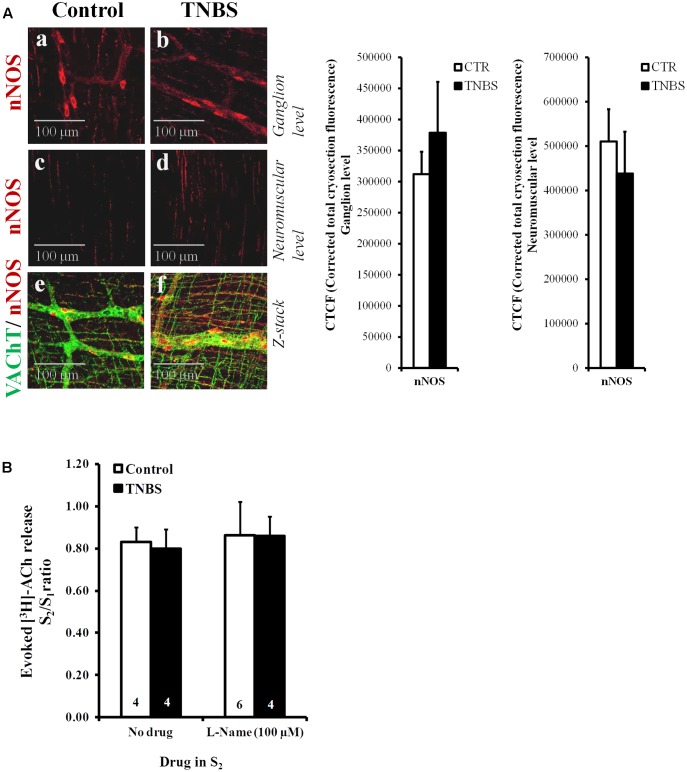
**(A)** Confocal micrographs of whole-mount preparations of the longitudinal muscle-myenteric plexus of the ileum from control and TNBS-treated rats taken at the myenteric ganglion level **(a,b)** and at the longitudinal smooth muscle layer **(c,d)**. Shown is the immunoreactivity against neuronal NOS (nNOS). Z-stacks presented in panels **(e)** and **(f)** show that nNOS-positive nitrergic nerve fibers (red) are adjacent, but do not co-localize, with VAChT immunoreactivity (green). Images are representative of at least four individuals per group. Scale bar = 100 μM. Bar charts at the right hand-side panel represent mean ± SEM of corrected total cryosection fluorescence (CTCF) nNOS staining detected at the ganglion level and at the neuromuscular region. **(B)** Effect of the nitric oxide synthase (NOS) inhibitor, L-Name (100 μM), on [^3^H]ACh released from stimulated myenteric neurons of the ileum of control and TNBS-treated rats. After loading and washout periods, [^3^H]ACh release was elicited by two trains (S_1_ and S_2_) of electrical field stimulation, each consisting of 200 pulses delivered at a 5 Hz frequency. L-Name (100 μM) was applied 8 min before S_2_. The ordinates are changes in S_2_/S_1_ ratios compared to the S_2_/S_1_ ratio obtained without addition of any drug. The data are means ± SEM of four to six individuals.

The lack of obvious changes in the density of VAChT-positive cholinergic nerve fibers, prompted us to investigate whether cholinergic nerve hypoactivity had any relationship with increased volume inhibitory neurotransmission operated by NO, which can be released from neighboring nitrergic nerves, enteric glia cells and/or infiltrating immunocytes (MacEachern et al., 2015; see also **Figure 4A**). This was hypothesized because up-regulation of nitric oxide synthase (NOS) activity and enhancement of NO-mediated inhibitory neurotransmission were demonstrated in chronic inflammatory bowel diseases (Miller et al., 1993). **Figure 4B** shows that inhibition of NOS activity with L-NAME (100 μM) had no effect on evoked [^3^H]ACh release from the LM-MP of the ileum of control and TNBS-treated rats, indicating that NO does not mediate inhibition of [^3^H]ACh release in TNBS-induced ileitis. Quantification of neuronal NOS-immunoreactivity in the myenteric plexus of the ileum of TNBS-treated animals was not significantly (*P* < 0.05) different from control rats, both at the ganglion level and at the neuromuscular region (**Figure 4A** and adjacent bar charts).

### Post-inflammatory Shift from Neuronal to Non-neuronal Control of Evoked [^3^H]ACh Release from Stimulated Myenteric Neurons

**FIGURE 5:**
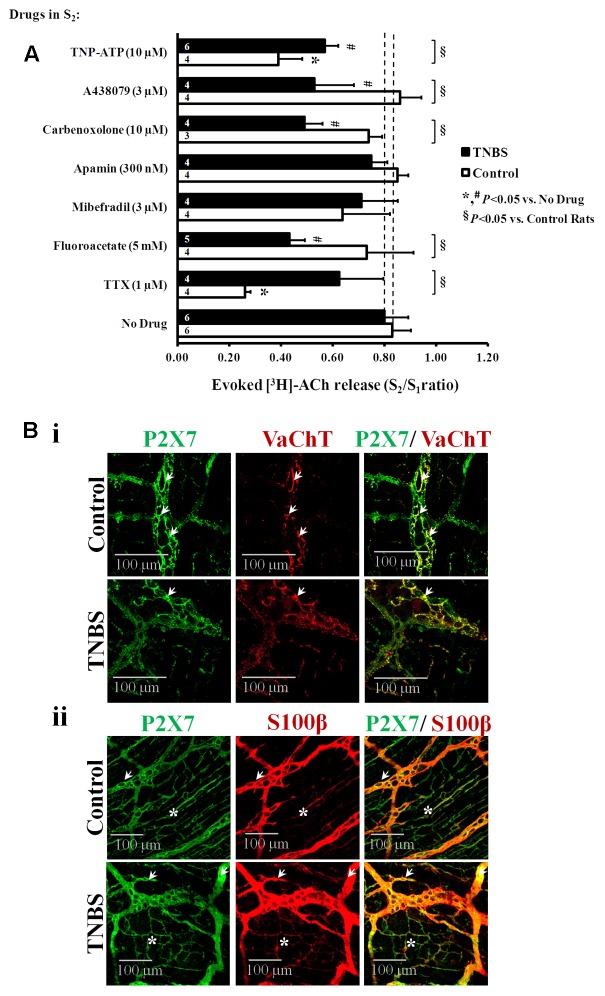
**(A)** Effects of TTX (1 μM, a nerve action potential blocker), sodium fluoroacetate (5 mM, a glial cell metabolism uncoupler), mibefradil (3 μM, an inhibitor of Ca_v_3 (T-type) channels existing in ICCs), apamin (0.3 μM, an inhibitor of small-conductance KCa2 (SK3) channels characteristic of FLCs), carbenoxolone (10 μM, a pannexin-1 inhibitor), A438079 (3 μM, a P2X7 receptor antagonist) and TNP-ATP (10 μM, a blocker of ionotropic P2X2 and P2X2/3 receptors) on electrically evoked [^3^H]-ACh release from the LM-MP of the rat ileum of control and TNBS-treated rats. After loading and washout periods, [^3^H]ACh release was elicited by two trains (S_1_ and S_2_) of electrical field stimulation, each consisting of 200 pulses delivered at a 5 Hz frequency. Drugs were applied 8 min before S_2_. Abscissa is changes in S_2_/S_1_ ratios compared to the S_2_/S_1_ ratio obtained without addition of any drug. The data are means ± SEM of three to six individuals. ^∗,#^*P* < 0.05 (unpaired Student’s *t*-test with Welch correction) represent significant differences from the situation where no drugs were added to control and TNBS-treated preparations, respectively (dashed vertical lines); ^§^ *P* < 0.05 (unpaired Student’s *t*-test with Welch correction) represent significant differences from control animals. Confocal micrographs of whole-mount preparations of the longitudinal muscle-myenteric plexus of the ileum from control and TNBS-treated rats. Shown is the P2X7 receptor immunoreactivity in preparations also stained against the vesicular ACh transporter (VAChT, **Bi**) and the enteric glial cell marker (S100β, **Bii**). The P2X7 receptor co-localizes partially with VAChT at the ganglion level (**Bi**, white arrows); a more extensive co-localization exists between the P2X7 receptor and the glial cell marker, S100β **(Bii)**, both at myenteric ganglia (white arrows) and at the neuromuscular layer (asteriks) of control and TNBS-treated preparations. Z-stack images are representative of at least four animals per group. Scale bar = 100 μm.

**Figure 5A** shows that [^3^H]ACh release from electrical-stimulated ileal myenteric neurons of healthy rats was almost prevented (*P* < 0.05) by the blockage of nerve action potentials with tetrodotoxin (TTX, 1 μM). This confirms our previous observations, where we also demonstrated that [^3^H]ACh excoytosis from depolarized myenteric nerve terminals depended on Ca^2+^ influx through voltage-sensitive channels (Duarte-Araújo et al., 2004a,b; Correia-de-Sá et al., 2006). Interestingly, inhibition of electrically driven [^3^H]ACh outflow from TNBS-treated preparations was much less sensitive to TTX (1 μM) than control preparations (**Figure 5A**), but transmitter release was still fully prevented (*P* < 0.05) in Ca^2+^-free conditions, i.e., the S_2_/S_1_ ratio decreased from 0.80 ± 0.09 (*n* = 6) to 0.13 ± 0.01 (*n* = 4) upon removing Ca^2+^ from the Tyrode’s solution. Thus, the results suggest that [^3^H]ACh outflow in TNBS-induced ileitis is likely due to exocytosis triggered via direct axonal activation by a non-neuronal released activator (see e.g., Broadhead et al., 2012; Gulbransen et al., 2012).

Under the present experimental conditions, the influence of smooth muscle contractions was ruled out since the Ca_v_1 (L-type) channel blocker, nifedipine (1 μM), depressed ACh-induced contractions of the longitudinal muscle without affecting the release of [^3^H]ACh from stimulated LM-MP of both control (Correia-de-Sá et al., 2006) and TNBS-treated rats (unpublished observations). Likewise, we discarded the participation of ICCs and fibroblasts-like cells (FLCs) in the regulation of evoked [^3^H]ACh release in both animal groups, because the transmitter release was not (*P* > 0.05) affected when the experiments were performed in the presence of selective inhibitors of ICCs and FLCs activity (**Figure 5A**), namely mibefradil (3 μM) and apamin (0.3 μM) which block specifically voltage-sensitive Ca_v_3 (T-type) and small-conductance K_Ca_2 (SK3) channels that are characteristic of these cells, respectively (Fujita et al., 2003).

Next, we tested whether the gliotoxin, sodium fluoroacetate (5 mM) (MacEachern et al., 2015), could affect [^3^H]ACh release from stimulated myenteric neurons. **Figure 5A**, shows that while sodium fluoroacetate (5 mM) was unable to change transmitter release in preparations from healthy rats, it significantly (*P* < 0.05) decreased evoked [^3^H]ACh release from myenteric neurons of TNBS-treated animals. These results led us to the hypothesis that the cholinergic tone is kept to a minimum due to the release of an excitatory gliotransmitter from proliferating enteric glial cells in post-inflammatory ileitis.

### ATP May Be the Putative Excitatory Gliotransmitter Responsible for Keeping the Cholinergic Tone in Post-inflammatory Ileitis

The nature of the putative excitatory gliotransmitter regulating ACh release from inflamed myenteric neurons is uncertain. Considering our previous observations that (1) ATP can be released from both neuronal and non-neuronal cells, and that (2) it can increase the release of [^3^H]ACh from resting myenteric nerve terminals through the activation of ionotropic P2X receptors in the presence of TTX (Duarte-Araújo et al., 2009), we designed experiments to test if endogenous ATP could play any role in the regulation of ACh release in TNBS-induced ileitis.

Recently, our group demonstrated that ATP can be released from non-neuronal cells through hemichannels containing pannexin-1 and this mechanism can lead to a “vicious cycle” where ATP can induce the release of ATP via the activation of ionotropic P2X and metabotropic P2Y receptors under normal and pathological conditions (Pinheiro et al., 2013; Noronha-Matos et al., 2014; Timóteo et al., 2014; Certal et al., 2015; Silva et al., 2015). High extracellular ATP levels, such as those detected after an inflammatory insult, can stimulate low-affinity P2X7 receptors, which often but not exclusively couple to pannexin-1 to promote ATP outflow, via the pannexin-1 hemichannel or the P2X7 receptor pore (Gulbransen and Sharkey, 2012; Diezmos et al., 2013). This prompted us to investigate the role of pannexin-1 hemichannels and P2X7 receptors on [^3^H]ACh release from stimulated LM-MP preparations of control and TNBS-treated rats using specific inhibitors.

**Figure 5A**, shows that selective blockage of pannexin-1 hemichannels and P2X7 receptors respectively with carbenoxolone (10 μM) and A438079 (3 μM) significantly (*P* < 0.05) decreased [^3^H]ACh release from myenteric neurons of TNBS-treated rats, but not of their control littermates. The magnitude of the inhibitory effects on transmitter release produced by carbenoxolone (10 μM) and A438079 (3 μM) was about the same of that observed upon blocking glial cells activity with sodium fluoroacetate (5 mM) (**Figure 5A**). Notwithstanding the fact that P2X7 receptors are present in VAChT-positive cholinergic nerve terminals in myenteric ganglia (**Figure 5Bi**), the distribution of the P2X7 immunoreactivity in the myenteric plexus of both groups of animals accompanies that of the glial cell marker, S100β (**Figure 5Bii**). Co-localization of the P2X7 receptor and the S100β glial marker has been demonstrated before in the rat myenteric plexus (Vanderwinden et al., 2003; Gulbransen and Sharkey, 2012). This feature was confirmed and expanded to post-inflammatory ileitis in our experimental settings as demonstrated by the elevated scores of the staining overlap (0.706 ± 0.059 and 0.703 ± 0.001 in control and TNBS-treated rats, respectively; 4 animals per group, *P* < 0.01) and the Pearson’s coefficient (ρ = 0.674 ± 0.074 and ρ = 0.665 ± 0.006 in control and TNBS-treated rats, respectively; 4 animals per group, *P* < 0.01) obtained by merging the two fluorescence channels (yellow staining; see **Figure 5Bii**).

**FIGURE 6:**
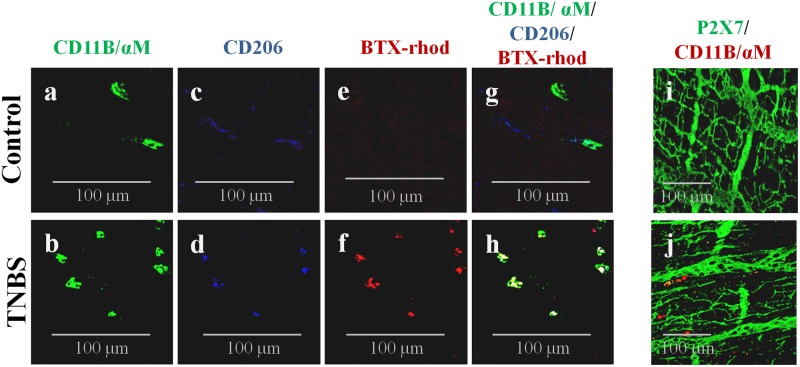
CD11B-immunoreactive inflammatory cells surrounding myenteric ganglia and infiltrating the intramuscular layer of TNBS-treated rats also stain positively against CD206 (a cell marker of anti-inflammatory M2 macrophages) and tetramethylrhodamine-conjugated α-bungarotoxin (BTX-rhod) identifying α7 subunit-containing nicotinic receptors both in Control **(a,c,e,g)** and TNBS-treated **(b,d,f,h)** rats. Confocal micrographs of whole-mount preparations of the longitudinal muscle-myenteric plexus of the ileum of Control **(i)** and TNBS-treated **(j)** rats show that CD11B-positive cells do not express the P2X7 receptor. Images are representative of at least four animals per group. Scale bar = 100 μm.

We discarded the presence of P2X7 receptors in CD11B^+^/ChAT^+^ inflammatory cells surrounding myenteric ganglia and/or infiltrating the intramuscular layer of the ileum of TNBS-treated rats (**Figures 6i,j**), thus eliminating the contribution of P2X7 receptors to TTX-resistant ACh release from these cells. Notwithstanding this, our data show that a subset of CD11B-labeled cells present in the myenteric plexus of TNBS-treated rats are also positive to the scavenger mannose C-type receptor, CD206, and to tetramethylrhodamine-conjugated α-bungarotoxin (BTX-rhod) (**Figures 6a–h**). Co-localization of CD11B and CD206 is reflected by increases in the staining overlap from 0.170 ± 0.028 in control animals to 0.431 ± 0.169 in TNBS-treated rats, while the same occurred concerning the Pearson’s coefficient, i.e., the ρ value increased from 0151 ± 0.027 in control animals to 0.411 ± 0.178 in TNBS-treated rats (4 animals per group, *P* < 0.01). A similar situation was verified when double immunolabelling CD11B or CD206 positive inflammatory cells with BTX-rhod. This labeling pattern suggests that CD11B-positive inflammatory cells next to myenteric neurons of TNBS-treated rats have an increasing proportion of anti-inflammatory macrophages of the M2 subtype (**Figures 6a–h**; Rõszer, 2015) carrying α7 nicotinic receptors, which cohabitate with a still significant amount of CD11B^+^/CD206^-^ cells (most probably monocytes-derived pro-inflammatory M1 macrophages). Activation of α7 subunit-containing nicotinic receptors by ACh in resident M2 macrophages modulates ATP-induced Ca^2+^ responses which play a key role in the gastrointestinal cholinergic anti-inflammatory pathway (Matteoli et al., 2014).

Murine enteric neurons express mainly P2X2 and P2X3 subunit-containing receptors (Galligan, 2002; Castelucci et al., 2002; Ren et al., 2003), which are responsible for ATP-induced Ca^2+^ transients (Ohta et al., 2005) and [^3^H]ACh overflow from resting myenteric neurons in the presence of TTX (Duarte-Araújo et al., 2009). Fast desensitizing homomeric P2X2 and/or heteromeric P2X2/3 receptor channels can be blocked by micromolar concentrations of trinitrophenyl-substituted nucleotides, especially TNP-ATP (Virginio et al., 1998). In control rats, TNP-ATP (10 μM) reduced the release of [^3^H]ACh by 53 ± 10% (*n* = 4), but its effect was significantly decreased to 17 ± 5% (*n* = 5, *P* < 0.05) in the ileum of TNBS-treated animals (**Figure 5A**). These findings raise the question about the purinoceptor subtype involved in ATP-induced transmitter release from myenteric neurons in post-inflammatory ileitis, a situation where the extracellular concentration of the nucleotide dramatically increases (Vieira et al., 2014). The low-affinity/slow-desensitizing P2X7 receptor is the most probable candidate, because (1) evoked [^3^H]ACh release was significantly attenuated by A438079 (3 μM) (**Figure 5A**), and (2) VAChT-positive cholinergic myenteric nerve terminals exhibit P2X7 receptor immunoreactivity (**Figure 5Bi**).

### Post-inflammatory Myenteric Glial Cells Release Higher Amounts of ATP

**FIGURE 7:**
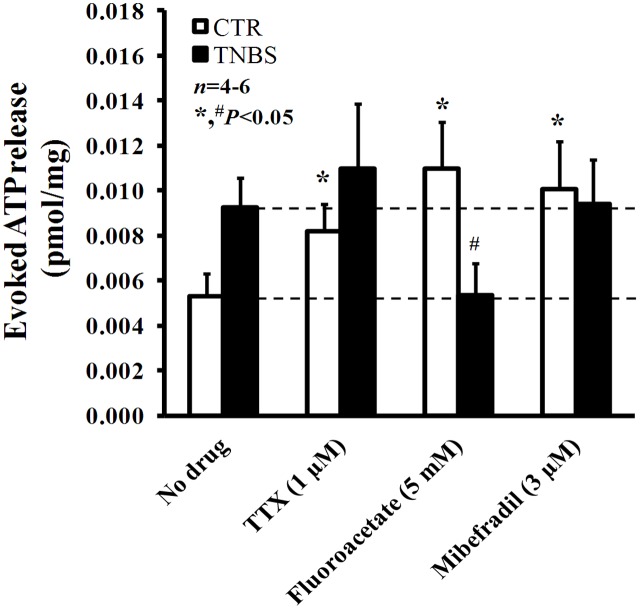
Effects of TTX (1 μM), sodium fluoroacetate (5 mM) and mibefradil (3 μM) on ATP release from stimulated longitudinal muscle-myenteric plexus preparations of the ileum from control and TNBS-treated rats. Drugs were in contact with the preparations for at least 15 min before stimulus application (5 Hz frequency, 3000 pulses of 1 ms duration). Samples from the incubation media were collected and analyzed using the luciferin-luciferase bioluminescence assay for ATP quantification (this figure); aliquots of the same samples were used in parallel to measure their content in adenosine (plus inosine) by HPLC with diode array detection (results in **Figure 8**). Ordinates represent stimulation-induced increases in ATP above baseline levels determined immediately before the stimulus application; results are expressed in pmol/mg of tissue. The data are means ± SEM of four to six animals. ^∗,#^*P* < 0.05 (unpaired Student’s *t*-test with Welch correction) represent significant differences from the amount of ATP released in the absence of any drug added to preparations from control and TNBS-treated animals (dashed horizontal lines), respectively.

In a previous study from our group, we demonstrated that ATP is released in higher amounts from electrically stimulated TNBS-treated preparations than in control tissues (Vieira et al., 2014). High post-inflammatory ATP levels are comparable to those obtained in control LM-MP preparations submitted to blockage of action potentials generation, of glial cells metabolism and of ICCs activation respectively with TTX (1 μM), sodium fluoroacetate (5 mM) and mibefradil (3 μM) (**Figure 7**). These findings indicate that all these cells contribute to keep low extracellular ATP levels in normal physiological conditions. However, this scenario changes considerably in TNBS-induced ileitis. From the inhibitors used to target specifically the three resident cell types in the myenteric plexus, only sodium fluoroacetate (5 mM) was able to decrease significantly (*P* < 0.05) the release of ATP in post-inflammatory ileitis, while TTX (1 μM) and mibefradil (3 μM) were both ineffective (**Figure 7**).

This suggests that increased overflow of ATP after an inflammatory insult may possibly result from activation of proliferating glial cells. Interestingly, the pharmacology affecting ATP overflow from stimulated ileal preparations of TNBS-treated rats (**Figure 7**) looks like that verified by measuring the release of [^3^H]ACh (**Figure 5A**), further strengthening our hypothesis that ATP may be the putative excitatory gliotransmitter responsible for keeping to a minimum the cholinergic tone in TNBS-induced ileitis.

Moreover, blockade of pannexin-1 hemichannels with carbenoxolone (10 μM) and P2X7 receptors with A438079 (3 μM) significantly (*P* < 0.05) decreased ATP overflow in TNBS-treated rats; while carbenoxolone (10 μM) was more active in decreasing the resting release of the nucleotide (from 5.1 ± 0.3 to 2.8 ± 0.4 fmol/mg, *n* = 4), A438079 (3 μM) depressed the release of ATP induced by electrical stimulation to the control level (from 9.2 ± 0.8 to 5.1 ± 0.6 fmol/mg, *n* = 4). These results suggest that pannexin-1 hemichannels may drive ATP release even during resting conditions, while ATP-induced ATP release via the activation of low affinity P2X7 receptors requires high extracellular levels of the nucleotide that might be favored by electrical stimulation of the tissue.

### Deficient Extracellular Adenosine Accumulation in Post-inflammatory Ileitis Parallels the Loss of ICCs

**FIGURE 8:**
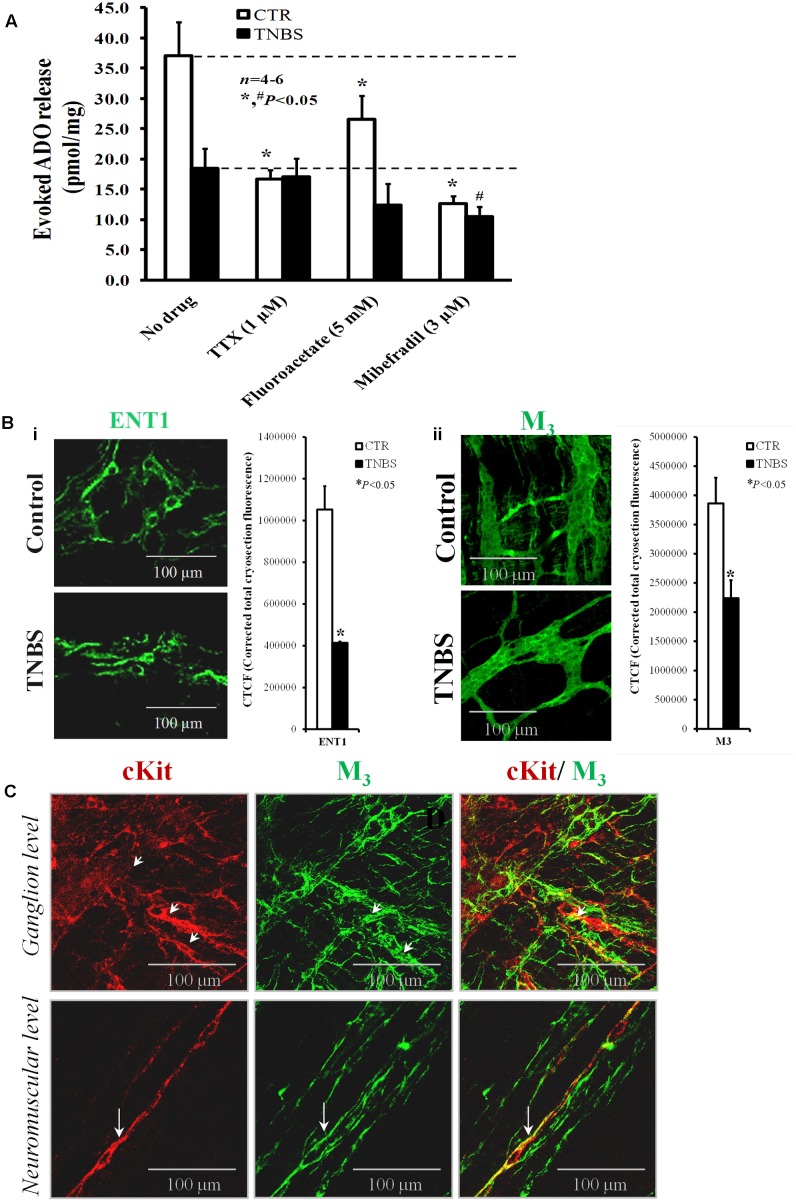
**(A)** Shows the effects of TTX (1 μM), sodium fluoroacetate (5 mM) and mibefradil (3 μM) on evoked adenosine (plus inosine) release from stimulated LM-MP ileal preparations of control and TNBS-treated rats. Drugs were in contact with the preparations for at least 15 min before EFS. Samples from the incubation media were collected and analyzed by HPLC with diode array detection to evaluate their content in adenine nucleosides (see **Supplementary Figure S1**); aliquots of the same samples were tested in parallel using the luciferin-luciferase bioluminescence assay for ATP quantification (results in **Figure 7**). Ordinates represent stimulation-induced increases in adenosine (plus inosine) above baseline levels determined immediately before EFS; results are expressed in pmol/mg of tissue. The data are means ± SEM of four to six individuals. ^∗,#^*P* < 0.05 (unpaired Student’s *t*-test with Welch correction) represent significant differences from the amount of adenosine (plus inosine) released in the absence of any drug added to preparations from control and TNBS-treated animals (dashed horizontal lines), respectively. **(B)** Shown are confocal micrographs of whole-mount preparations of the longitudinal muscle-myenteric plexus of the ileum from control and TNBS-treated rats stained against the equilibrative nucleoside transporter 1 (ENT1) (i) and the muscarinic M_3_ receptor. The staining pattern of both antibodies resembles that obtained for anoctamine-1 (Ano-1)-positive interstitial cells at the ganglion level shown **Figure 2**. Images are representative of at least four individuals per group. Scale bar = 100 μM. Bar charts at the right hand-side panels represents mean ± SEM of corrected total cryosection fluorescence (CTCF) of ENT-1 and muscarinic M_3_ antibodies staining, respectively. ^∗^*P* < 0.05 (unpaired Student’s *t*-test with Welch correction) represent significant differences from control animals. **(C)** Shown is confocal micrographs of whole-mount preparations of the longitudinal muscle-myenteric plexus (LM-MP) of the ileum of control rats. Please note that the majority of c-Kit positive interstitial cells of Cajal (ICCs, red) at the neuromuscular region are endowed with muscarinic M_3_ receptors (green) (long arrows); although co-localization of c-kit (red) and muscarinic M_3_ receptor (green) is still apparent at the myenteric ganglion level (small arrows), most of the cells express only one of these markers. Yellow staining denotes co-localization when overlaying the green and red confocal channels.

Although adenosine may be released from activated inflammatory cells (Marquardt et al., 1984) in the vicinity of myenteric neurons (Bogers et al., 2000), the loss of adenosine neuromodulatory control of evoked [^3^H]ACh release in TNBS-induced ileitis detected in a previous report results mainly from deficient accumulation of the nucleoside at the myenteric synapse (Vieira et al., 2014); this was verified despite the increase in ATP content of the same samples. Uncoupling between ATP overflow and adenosine levels in post-inflammatory ileitis was ascribed to feed-forward inhibition of ecto-5′-nucleotidase/CD73 and upregulation of adenosine deaminase. Here, we decided to evaluate the putative contribution of inflammation-induced cell density changes in the myenteric plexus to adenosine deficiency. To this end, we measured in parallel to the ATP levels the extracellular concentration of adenosine and of its deamination metabolite, inosine, by HPLC (with diode array detection) immediately before and after electrical stimulation of LM-MP preparations of both control and TNBS-treated rats in the presence of cell-specific activity inhibitors. **Figure 8A** shows that the amounts of adenosine (plus inosine) released into the extracellular fluid following stimulation of the LM-MP are much higher in healthy controls than in TNBS-treated rats. It is worth to note that the amount of adenosine (plus inosine) accumulated in the LM-MP of healthy rats following electrical field stimulation was 7,000-fold higher than the ATP concentration in the same collected samples. It is also important to notice that we were unable to detect β-NAD^+^ and/or cyclic AMP in our collected samples, neither before nor after electrical stimulation of the preparations; this was verified despite our chromatographic system is suitable to detect standards of these putative adenosine precursors in the same (picomolar) concentration range (data not shown). In view of this, stoichiometric conversion of ATP (or other released adenine nucleotide) into adenosine by ectonucleotidases can barely be considered a major source of the nucleoside in the myenteric plexus of the rat ileum (see e.g., Correia-de-Sá et al., 2006), thus confirming our previous suspicions that high amounts of adenosine may be released as such from neuronal and/or non-neuronal cells (Duarte-Araújo et al., 2004a).

Stimulus-evoked adenosine (plus inosine) release was partially dependent on neuronal activity in preparations from healthy rats, but not in the ileum of TNBS-treated animals. This was assumed because pre-treatment of the preparations with TTX (1 μM) reduced by about 50% the outflow of adenine nucleosides in control tissues with no effect on TNBS-treated preparations (**Figure 8A**). This means that the neuronal source of adenosine may be severely affected in TNBS-induced ileitis due to neuronal cells dysfunction (see above). Even though we considered unlikely that proliferating glial cells could contribute to adenosine deficits in the inflamed myenteric plexus of the rat ileum, we tested the effect of the glial cell metabolic uncoupler, sodium fluoroacetate (5 mM). Inhibition of glial cells metabolism caused a significant (*P* < 0.05), yet of smaller magnitude compared TTX, reduction of adenosine outflow from stimulated LM-MP preparations of control rats, whereas sodium fluoroacetate (5 mM) had only a minor effect (*P* > 0.05) in the myenteric plexus isolated from TNBS-treated rats (**Figure 8A**). The inhibitory action of sodium fluoroacetate (5 mM) was slightly more evident in healthy tissues than in TNBS-treated preparations, which is in agreement with the proposed uncoupling between ATP overflow and adenosine formation secondary to the inflammatory insult.

Prevention of smooth muscle contractions with the voltage-sensitive Ca_v_1 (L-type) channel inhibitor, nifedipine (5 μM), did not affect the release of adenosine (plus inosine) in both animal groups (Correia-de-Sá et al., 2006; Vieira et al., 2009, 2014). However, blockade of Ca_v_3 (T-type) channels expressed in ICCs with mibefradil (3 μM) significantly (*P* < 0.05) attenuated the release of adenosine (plus inosine) from both control and TNBS-treated animals. Taking into consideration previous findings from our laboratory demonstrating that the nucleoside transport inhibitor, dipyridamole (0.5 μM), decreased proportionally and by a similar amount the outflow of adenosine (plus inosine) to that obtained with mibefradil (3 μM) from both control and inflamed tissues (Vieira et al., 2014), it is very likely that adenosine accumulated in the ileum following an inflammatory insult originates predominantly from activated ICCs via a dipyridamole-sensitive equilibrative nucleoside transporter (ENT). The presence of ENT1-immunoreactivity in ICC-like cells located in the myenteric plexus of the ileum of control and TNBS-treated rats is shown in **Figure 8Bi**. The decrease in ENT1 immunoreactivity seems to parallel that of Ano-1 positive ICCs in the myenteric plexus of the ileum of TNBS-injected animals (see **Figures 2A**, **8Bi**).

In a previous study, we showed that adenosine outflow via dipyridamole-sensitive ENT-1 may be positively modulated by muscarinic M_3_ receptors in the LM-MP of the rat ileum leading to activation of facilitatory A_2A_ receptors on cholinergic nerve terminals (Vieira et al., 2009). Signals from myenteric motor nerves onto ICCs involve M_3_ receptors in mice intestine (Wang et al., 2003; So et al., 2009), indicating that a close relationship between enteric excitatory nerve terminals and intramuscular ICCs is fundamental to modulate cholinergic neurotransmission in the enteric nervous system (Wang et al., 2003). **Figure 8C** shows that muscarinic M_3_ receptors are located in a small subset of c-Kit positive ICCs in myenteric ganglia, but this proportion significantly increases in c-Kit expressing intramuscular ICCs. Similar results were obtained in murine small intestine and gastric fundus (Epperson et al., 2000; Lecci et al., 2002), with muscarinic M_3_ receptors staining outside c-Kit-labeled cells most probably located in enteric neurons. Interestingly, muscarinic M_3_ receptors immunoreactivity substantially decreased in extraganglionic myenteric cells of TNBS-injected animals compared to their control littermates (**Figure 8Bii**). This pattern resembles that obtained in micrographs stained with ENT1 and Ano-1 antibodies (see **Figures 2A**, **8Bi**, respectively). Unfortunately, we were unable to double label LM-MP preparations with ENT1 plus M_3_ and ENT-1 plus Ano-1 because the available antibodies were raised in the same host species (rabbit) (see **Table 1**); in addition, we felt technical difficulties when attempting to target ENT-1 in c-Kit-stained ICCs because the latter requires the use of a tissue fixative solution containing 50% acetone and 2% PFA in PBS, which revealed incompatible with the ENT-1 immunostaining.

## Discussion

### TNBS-Induced Ileitis Affects Cholinergic Function without Neuronal Cell Loss: A Model to Study Neuronal to Non-neuronal Cell Communication after Inflammatory Insults

A number of key cellular players may be involved in the reaction to inflammatory insults by the gastrointestinal system. These include inflammatory cells, such as granulocytes, lymphocytes, mast cells, monocytes and macrophages, which release numerous cytokines, pro-inflammatory peptides, neuroactive and neurotrophic factors, noxious compounds, inflammatory enzymes and other danger signaling molecules. Enteric plasticity comprises a wide range of structural and/or functional changes in enteric neurons, glial cells and interstitial cells (ICC/FLC). As a result of adaptive responses to different types of pathophysiological conditions, enteric neurons are able to rapidly change their structure, function and chemical phenotype in order to maintain homeostasis of gut functions. In contrast to long-term functional abnormalities in the activity of neuronal cells described by several authors (Stewart et al., 2003; Nurgali et al., 2007) and confirmed in the present study by denoting hypoactivity of cholinergic neurotransmission, we found no obvious modifications in the cellular density and distribution of specific neuronal cell markers (e.g., NF200, PGP 9.5, VAChT, nNOS) in TNBS-induced ileitis. In this sense, ileitis caused by TNBS differs from other chronic inflammatory disease models because it lacks the chronic inflammatory phase (Moreels et al., 2001) that is usually accompanied by neuronal cell loss (Sanovic et al., 1999; Linden et al., 2005; Venkataramana et al., 2015). Notwithstanding, this constrain may be turned into an advantage characteristic of this animal model if one wants to investigate the adaptive changes of neuronal to non-neuronal cells communication after a transient inflammatory insult, as we aimed in this work.

Results show that stimulated myenteric neurons release smaller amounts of [^3^H]ACh in the ileum of TNBS-treated rats compared to their control littermates, without evidence of any alteration in the density of VAChT-positive cholinergic nerve fibers in the myenteric plexus. Likewise, significant decreases in [^3^H]choline uptake, acetylcholine release and contractile responses to stimulation of enteric nerves have been observed in rats with colitis induced by TNBS (Poli et al., 2001). These results imply a functional loss, not corresponding to cellular depletion, of cholinergic neurotransmission in inflamed tissues (Collins et al., 1992a). The mechanism underlying attenuation of ACh release in the inflamed rat intestine has been a matter of debate in the literature, but so far there is no unifying theory given the contradictory findings when looking at different immune cell players and inflammatory mediators (e.g., Davis et al., 1998). This is so, even though evidences have been produced showing that cytokines may directly change neurotransmitters content and release (Collins et al., 1992b). This contention strengthens our hypothesis that functional and structural adaptations of resident non-neuronal myenteric cell populations of the rat ileum may play a relevant role in the mechanisms underlying downregulation of cholinergic neurotransmission in TNBS-induced ileitis.

### Post-inflammatory Depletion of ICCs Correlates with Adenosine Deficiency and Decreased Frequency of Spontaneous Contractions in the Ileum of TNBS-Treated Rats

Using immunofluorescence confocal microscopy, we show here that the LM-MP of the rat ileum becomes deficient in Ano-1 positive ICCs, both in the ganglion layer (myenteric stellate cells) and at the neuromuscular region (intramuscular spindle-shape cells). The same occurred regarding immunostaining against vimentin, which is an intermediate filament protein also present in FLCs. Porcher et al. (2002) demonstrated an almost complete abolition of interstitial cells in biopsy samples from patients with Crohn’s disease. In the more severe colitis rat model, muscularis resident macrophages have been implicated in the loss of ICCs detected 7-days after instillation of TNBS (Kinoshita et al., 2007). TNF-α secreted from classically activated M1 macrophages reduces the number of cultured ICCs, whereas the conditioned medium from M2 macrophages had no effect (Eisenman et al., 2017). Co-localization studies show here that despite the increasing proportion of CD11B^+^/CD206^+^/BTX-rhod^+^ anti-inflammatory M2 macrophages (see Rõszer, 2015) next to myenteric neurons of TNBS-treated rats, these cells cohabitate with a still important subset of CD11B^+^/CD206^-^ inflammatory cells, most probably composed of monocytes-derived pro-inflammatory M1 macrophages. This inflammatory cell pattern may be responsible for the loss of ICCs detected in post-inflammatory ileitis and may account to the decrease in the frequency of spontaneous contractions of LM-MP preparations of the ileum of TNBS-treated rats, as these cells have been implicated in the generation of electrical slow waves regulating the phasic contractile activity of the gastrointestinal smooth muscle (Kinoshita et al., 2007).

To our knowledge, this is the first report showing a relationship between the loss of ICCs in the myenteric plexus of the rat ileum following an inflammatory insult and depletion of extracellular adenosine compared to the normal physiological condition. This was concluded because the lowest extracellular adenosine levels in the ileum were detected upon blocking the activity of ICCs with the Ca_v_3 (T-type) channel inhibitor, mibefradil, both in control conditions and after the inflammatory insult with TNBS. Therefore, it is highly likely that this mechanism, together with the feed-forward inhibition of ecto-5′-nucleotidase/CD73 and upregulation of adenosine deaminase shown in our previous study (Vieira et al., 2014), may concur to prevent adenosine-mediated actions in post-inflammatory ileitis. Considering that, under normal physiological conditions, endogenous adenosine facilitates [^3^H]ACh release from stimulated myenteric neurons through the preferential activation of pre-junctional A_2A_ receptors (Duarte-Araújo et al., 2004a), one may speculate that cholinergic hypoactivity in TNBS-induced ileitis is dependent on the loss of adenosine A_2A_ receptor-mediated neurofacilitation. Although not explored in this study cholinergic nerve hypoactivity in TNBS-induced ileitis may also be due to reinforcement of the muscarinic M_2_-receptor-mediated pre-synaptic auto-inhibition (Takeuchi et al., 2005; Vieira et al., 2009). Yet, even if this mechanism is verified it may be cut-short by the resultant decline of [^3^H]ACh release from inflamed myenteric nerves. One must also not forget that neurons are an important source of extracellular adenosine in the ileum of healthy animals (Correia-de-Sá et al., 2006) and this resource may turn to be deficient accompanying inflammation-induced functional abnormalities of myenteric neurons, which cannot be compensated by the concurrent loss of interstitial cells.

### Depletion of ICCs Downregulates M_3_ Receptor-Mediated Adenosine Overflow via ENT1, Decreasing Facilitation of Cholinergic Neurotransmission via Pre-synaptic A_2A_ Receptors

Purinergic re-enforcement to maintain cholinergic neurotransmission during high enteric nerve activity or whenever endogenous levels of adenosine become elevated has been observed in hypoxia, inflammation and postoperative ileum (Milusheva et al., 1990; De Man et al., 2003; Kadowaki et al., 2003). In a previous study, we provided evidence suggesting that muscarinic M_3_ receptors activation by neuronally released ACh triggers a positive feedback loop leading to facilitation of transmitter release that is indirectly mediated by adenosine outflow and activation of pre-junctional A_2A_ receptors (Vieira et al., 2009). Yet, at that time we had no information regarding the cellular players of the tripartite myenteric neuromuscular synapse involved in this pathway. Nowadays, we learned that c-kit positive ICCs are endowed with muscarinic M_3_ receptors coupled to G_q/11_ (reviewed in Ward and Sanders, 2001; Goyal, 2013; see also **Figure 8**) and these receptors can be activated by inhibiting endogenous ACh breakdown with physostigmine resulting in adenosine overflow from the myenteric plexus of healthy rats (Vieira et al., 2009). Activation of phospholipase C (PLC) by muscarinic M_3_ receptors causes the formation of IP_3_ and DAG with subsequent recruitment of intracellular Ca^2+^ and protein kinase C activation, respectively (Koh and Rhee, 2013). Increases in intracellular Ca^2+^ can strengthen activation of protein kinase C, which subsequently stimulates adenosine outflow via the equilibrative transporter ENT1 (Coe et al., 2002). Increase in protein kinase C activity might also stimulate 5′-nucleotidase inside cells (Obata et al., 2001) and/or inhibit adenosine kinase (Sinclair et al., 2000), enhancing intracellular adenosine accumulation and the efflux of the nucleoside from the cells. These hypotheses were confirmed in the myenteric plexus of healthy rats incubated with the protein kinase C activator, phorbol 12-myristate 13-acetate, whereas extracellular adenosine accumulation was prevented by inhibiting ENT1 with dipyridamole (Vieira et al., 2009).

Using immunofluorescence confocal microscopy, we show here that the density of muscarinic M_3_ receptors decreased significantly in the myenteric plexus of the ileum of TNBS-treated rats. Downmodulation of M_3_ receptors-immunolabelling was more significant at the intramuscular level and paralleled the decrease in the immunostaining against ENT1 and Ano-1 in LM-MP preparations of rats injected with TNBS. In this context, our current vision is that the loss of ICCs expressing muscarinic M_3_ receptors in TNBS-induced ileitis contributes to reduce extracellular adenosine accumulation, breaking down the amplification loop initiated by ACh release from myenteric neurons that is mediated by muscarinic M_3_ receptors-induced adenosine overflow from ICCs, and concluded through the activation of facilitatory A_2A_ receptors on cholinergic nerve terminals. To our knowledge, this is the first time adenosine released from myenteric ICCs is implicated in functional cholinergic nerve abnormalities in post-inflammatory ileitis.

### Surplus ATP Released from Proliferating Glial Cells Contributes to Sustain Cholinergic Tonus at Minimum in TNBS-Induced Post-inflammatory Ileitis

While the findings discussed so far may justify the downregulation of cholinergic neurotransmission in TNBS-induced ileitis, they fail to explain why ACh release from inflamed myenteric neurons becomes partially resistant to blockade of nerve action potentials by TTX and the mechanism(s) underlying the TTX-resistant transmitter release that is low, but still significant, in inflamed preparations. The appearance of TTX-resistant sodium currents has been observed before in TNBS-induced ileitis (Stewart et al., 2003). Interestingly, the pharmacology concerning the modulation of [^3^H]ACh release from myenteric motor neurons of TNBS-treated rats is remarkably similar to that obtained when attempting to control ATP release in the same preparations. Notwithstanding the fact that inflammation-induced variations in the myenteric concentrations of ACh and ATP diverge (i.e., ACh decreases and ATP increases), the release of the two transmitters were significantly reduced in the presence of the gliotoxin, sodium fluoroacetate, but it was not affected by the neuronal activation blocker, TTX. These results led us to suggest that enteric glia cells are crucial to maintain ACh release from inflamed myenteric neurons and that ATP released from these cells may be the excitatory gliotransmitter responsible for keeping cholinergic tonus at minimum in TNBS-induced ileitis.

As a matter of fact, we observed increases in GFAP- and S100β-immunoreactivities in TNBS-induced ileitis suggesting that the inflammatory insult may stimulate enteric glial cells proliferation (Bradley et al., 1997) and/or activate the expression/synthesis of glial protein cell markers (Cabarrocas et al., 2003). The release of cytokines and growth factors from inflammatory and immune cells may promote glial cells proliferation (Fields and Burnstock, 2006). On the other hand, enteric glial cells may secrete high amounts of neurotrophic factors, like NGF and GDNF, which may change the neurochemical code and content of neurotransmitters in myenteric neurons, even if they do not vary in terms of density (Von Boyen and Steinkamp, 2010). Enteric glia cells may also be a source of pro-inflammatory cytokines, such as IL-6 and IL-1β (Stoffels et al., 2014), with the latter being associated with the suppression of ACh release from the myenteric plexus (Van Assche and Collins, 1996). However, if this were the case in TNBS-induced ileitis, blockade of enteric glia cells metabolism would enhance, rather than decrease, evoked [^3^H]ACh release from myenteric motor neurons. Moreover, enteric glia contain neurotransmitter precursors, uptake and degrade neuroactive substances, express neurotransmitter receptors and provide neurosupporting actions (Vasina et al., 2006). Regarding recovery from inflammatory adaptive cell changes, it appears that enteric glial dysfunction does not persist after the resolution of intestinal inflammation in TNBS-treated animals, indicating that attempts to correct glial cells dysfunction may be restricted to the course of inflammatory adaptations (MacEachern et al., 2015).

The function of enteric glial cells is intimately tied to the levels of purines and membrane-bound ectonucleotidases, which regulate the availability of P2 receptor ligands (Braun et al., 2004; Lavoie et al., 2011). Data from this study suggest that surplus ATP released by the myenteric plexus after an inflammatory insult may originate from proliferating glial cells, because ATP accumulation was reduced to control levels in the presence of the specific glial metabolic uncoupler, sodium fluoroacetate. ATP released from glial cells, acting most likely via pre-junctional homodimer P2X2 or heterodimer P2X2/3 receptors sensitive to TNP-ATP, may trigger the release of ACh directly from cholinergic nerve terminals (Galligan, 2002; Castelucci et al., 2002; Ren et al., 2003; Ohta et al., 2005; Duarte-Araújo et al., 2009; see also **Figure 5A**). Ionotropic P2X receptor subtypes are Ca^2+^ permeable non-selective cation channels (also permeable to Na^+^), which can also inhibit membrane potassium conductance in enteric neurons (Barajas-López et al., 1996). Via these mechanisms, ATP can cause membrane depolarization leading to a secondary opening of voltage-gated Ca^2+^ channels and to transmitter exocytosis in the absence of action potential generation (Duarte-Araújo et al., 2009; see also Barthó et al., 2006), thus contributing to the maintenance of the cholinergic tone within a certain extent. Redistribution of NTPDase2, but not NTPDase3 from ganglia to the neuromuscular region leads to preferential ADP accumulation from released ATP, particularly when extracellular ATP levels reach the V_max_ for NTPDase3 (Duarte-Araújo et al., 2009) like that occurring in inflamed neuromuscular synapses (Vieira et al., 2014). Inflammation-induced increase in the expression of NTPDase2 in intramuscular glia may also contribute to restrain nerve-evoked ACh release in TNBS-induced ileitis due to the activation of ADP-sensitive inhibitory P2Y_1_ receptors in myenteric nerve terminals (Duarte-Araújo et al., 2009), yet this hypothesis needs further experimental confirmation.

### The Post-inflammatory Shift from Neuronal to Non-neuronal ACh Release Involves Ionotropic P2X7 Receptors and ATP Release via Pannexin-1 Hemichannels from Proliferating Glial Cells

Next we attempted to investigate the mechanism underlying the control shift from neuronal to non-neuronal of ACh release in TNBS-induced ileitis. Results show that evoked ACh release from inflamed myenteric neurons was attenuated by blocking the P2X7 receptor-pannexin-1 pathway respectively with A438079 and carbenoxolone in a similar manner to that verified with sodium fluoroacetate. Interestingly, the P2X7 receptor immunoreactivity in the myenteric plexus of the rat ileum is remarkably similar to that obtained for the glial cell marker, S100β, indicating that they may co-localize as shown previously (Vanderwinden et al., 2003; Gulbransen and Sharkey, 2012). Despite this, the P2X7 receptor is also expressed in neuronal soma and nerve terminals, but it is absent from smooth muscle fibers, interstitial cells and anti-inflammatory M2 macrophages (CD11B^+^/CD206^+^cells).

The ionotropic P2X7 receptor is a non-selective cation channel allowing Na^+^ and Ca^2+^ influx and K^+^ efflux in the presence of high-micromolar ATP concentrations (Morandini et al., 2014). This receptor has been largely associated with inflammatory diseases, including inflammatory bowel diseases (Diezmos et al., 2013), and it can signal through caspase-1 to cause the production of pro-inflammatory IL-1β and IL-18 (Kurashima et al., 2012), as well as TNF-α and nitric oxide (Alves et al., 2013), from inflammatory cells. Prophylactic systemic P2X7 receptor blockade attenuates the severity of mucosal damage, lowers macrophage and T-cells infiltration, and prevents the production of pro-inflammatory cytokines like TNF-α and IL-1β (with no changes detected in anti-inflammatory TGF-β and IL-10) secondary to experimental colitis in rats (Marques et al., 2014). This information is supported by our findings showing that resident anti-inflammatory M2 macrophages (CD11B^+^/CD206^+^cells) in the myenteric plexus of the rat ileum do not express P2X7 receptors 7 days after the inflammatory insult. The corollary of this finding may be that P2X7-mediated pro-inflammatory actions in inflammatory bowel diseases are undertaken by monocyte-derived macrophages polarized toward the M1 phenotype (CD11B^+^/CD206^-^ cells). Macrophages of the M1 subtype overexpressing the P2X7 receptor were found in the inflamed mucosa and have been implicated in the pathogenesis of Crohn’s disease in human patients (Neves et al., 2014). These cells usually contribute to initiate and sustain inflammation through the release of large amounts of cytokines (e.g., IL-1β, TNF-α, IL-12, IL-18, and IL-23) and NO from L-arginine, whereas anti-inflammatory M2 macrophages are most often involved in phagocytic activity, immune confinement and tissue remodeling during the healing phase of inflammatory insults. It also became clear from our data that maintenance of the non-neuronal cholinergic tone attributed to activation of P2X7 receptors in post-inflammatory ileitis cannot be due to ACh release from CD11B^+^/CD206^+^/ChAT^+^ inflammatory cells infiltrating the myenteric plexus because these cells do not express this receptor.

Pannexin-1 is also expressed in a variety of immune cells and is required for caspase-1 cleavage in response to P2X7 receptor activation (Pelegrin and Surprenant, 2006). Downregulation of pannexin-1 is associated with pro-inflammatory responses, whereas the opposite promotes anti-inflammatory reactions (Sáez et al., 2014). Pannexin-1, along with pannexin-2, is expressed in all layers of the human colon, including the mucosa, muscularis mucosa, submucosa and muscularis externa, where it is found preferentially in enteric ganglia, but also in the endothelium of blood vessels, epithelial cells and goblet cells (Diezmos et al., 2013; Maes et al., 2015). The P2X7 receptor and pannexin-1 hemichannels form signaling complexes in several cell types and both structures may concur to release ATP from non-neuronal cells under physiological and pathological conditions. Interestingly, the selective P2X7 receptor antagonist, A438079, and the specific pannexin-1 inhibitor, carbenoxolone, did not modify evoked ACh release in the ileum of healthy rats where myenteric ATP levels are low, but they significantly decreased [^3^H]ACh overflow from preparations of TNBS-treated rats in conditions where extracellular ATP release from proliferating glial cells is assumed to be high. It has also been shown that activation of the P2X7 receptor in enteric neurons may elicit ATP release through pannexin-1 hemichannels and this may act as a danger signal for neurons (Gulbransen et al., 2012). However, this mechanism is unlikely to modulate [^3^H]ACh outflow in post-inflammatory ileitis because no substantial neuronal loss was observed in our experimental conditions and the transmitter release in TNBS-induced ileitis was TTX-resistant, yet fluoroacetate-sensitive, indicating a dominant participation of enteric glial cells.

### Pivotal Role of Enteric Glia and ICCs to Maintain the Integrity of Gut Function as Part of a General Strategy to Respond to GI Tract Disorders Involving Purinergic Signaling

It has been proposed that enteric glial cells may play a critical role in maintaining the integrity of the bowel and that their loss or dysfunction might contribute to cellular mechanisms of inflammatory bowel diseases. A fulminating and fatal jejuno-ileitis is produced 7–14 days after ablation of enteric glia in adult transgenic mice, with almost none effects in other parts of the GI tract including the colon (Bush et al., 1998). It remains, however, to be explored whether deficits in ATP outflow from deficient glial cells play a role in triggering this disease process. This would be clinically relevant, since we predict here that the nucleotide may be an important gliotransmitter responsible for sustaining cholinergic tone after an inflammatory insult and this might contribute to avoid degeneration of myenteric neurons as already proved for glutamate in the CNS (Bush et al., 1999). In this context, the vasoactive properties of released adenine nucleotides and nucleosides may also contribute to microvascular disturbances and certain changes resembling ischemic bowel (see e.g., Mendes et al., 2015).

**FIGURE 9:**
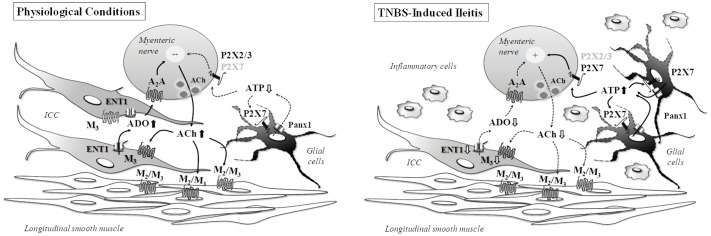
Inflammation-induced cholinergic hypoactivity is deeply dependent on the purinergic shift from a preferential adenosinergic tone under physiological conditions to a more prevalent ATP-mediated control of ACh release in TNBS-induced ileitis. The post-inflammatory loss of ICCs correlates with adenosine deficiency in the ileum of TNBS-treated rats breaking down facilitation of cholinergic neurotransmission operated by muscarinic M_3_ receptors-induced adenosine overflow from ICCs via ENT-1 and activation of pre-junctional facilitatory A_2A_ receptors. Adenosine depletion contrasts with the excess of ATP released from proliferating glial cells after the inflammatory insult and may also be explained by deficient formation from release adenine nucleotides by NTPDase cascade redistribution and excess of catabolism by adenosine deaminase (ADA) overproduction in inflammatory tissues (see Vieira et al., 2014). Extracellular ATP accumulation contributes to maintain the cholinergic tone at a minimum through the activation of high affinity / fast desensitizing pre-junctional P2X2 and/or P2X2/3 receptors. Inflammatory-induced neuronal to non-neuronal shift of ACh release is also intimately tight to a mechanism involving low affinity / slow desensitizing ionotropic P2X7 receptors and surplus ATP release via pannexin-1 hemichannels from proliferating glial cells. This scenario admits that post-inflammatory ileitis recapitulates an ancestral mechanism by which ATP released by glial cells directly (via ionotropic P2X receptors) stimulates ACh release from cholinergic nerve terminals even when these cells are disturbed and unable to generate normal action potentials. ACh: acetylcholine; ADO: adenosine; ENT1: equilibrative nucleoside transporter 1; ICC: interstitial cells of Cajal; Panx1: pannexin-1 hemichannels. Solid arrows indicate preferential mechanisms, while dotted arrows are assigned to deficient paths.

Thus, similarities in the appearance of end-stage pathology in various bowel conditions (e.g., inflammation, hypoxia/ischemia, diabetes) suggest that the gut may have limited ways of responding when it is defective (Bush et al., 1998). For this reason it is important to identify single events that are able to trigger complex pathological cascades. Our working hypothesis in this study was that inflammation-induced cell density changes in the myenteric plexus may unbalance the release of purines and, thus, their influence on ACh release from stimulated enteric nerves. We clearly demonstrated that inflammation-induced cholinergic hypoactivity is deeply dependent on the purinergic shift from a preferential adenosinergic tone under physiological conditions to a more prevalent ATP-mediated control of ACh release in TNBS-induced ileitis (**Figure 9**). The adenosine neuromodulation deficit parallels the partial loss of ICCs population in the inflamed myenteric plexus and contributes to explain the brake of the cholinergic amplification loop operated by facilitatory muscarinic M_3_ and adenosine A_2A_ receptors, which is responsible for keeping the safety margin of myenteric neuromuscular transmission under stressful conditions. On the other hand, proliferating and/or reactive enteric glial cells markedly influence the amount of ATP at the inflamed myenteric synapse by creating a vicious cycle that promote the release of the nucleotide through a mechanism involving the P2X7 receptor/pannexin-1 pathway. Data suggest that ATP may be the excitatory gliotransmitter responsible for keeping the cholinergic tone above a critical minimum in post-inflammatory ileitis. Inflammation may, thus, recapitulate an ancestral mechanism by which ATP released by glial cells directly (via ionotropic P2X receptors) stimulates ACh release from cholinergic nerve terminals even when these cells are disturbed and unable to generate normal action potentials.

## Conclusion

This study provides a deeper understanding of the pathophysiological impact of purines in the communication between non-neuronal cells and neurons underlying the dysregulation of cholinergic neurotransmission in post-inflammatory ileitis. Data suggest that the purinergic cascade (e.g., ATP release mechanisms, ecto-nucleotidase enzymes and purinoceptors activation) may offer novel therapeutic targets to overcome long-term cholinergic neurotransmission deficits affecting motility in inflammatory bowel diseases.

## Author Contributions

CV, FF, MTMC, IS, PM, and PC-dS contributed significantly for the experimental design, data acquisition, and interpretation of the results obtained; CV carried out surgical procedures and monitored postoperative animal welfare; CV, IS, and PM performed *in vitro* myographic recordings and experiments to measure ACh, ATP and adenosine release; CV and MTMC quantified adenine nucleotides and nucleosides by HPLC; CV and FF performed immunofluorescence staining and confocal microscopy observations; CV and PC-dS drafted and revised the manuscript; PC-dS supervised the project in all its dimensions; all authors approved the submitted version of the manuscript and agreed that all aspects of the work are accurate.

## Conflict of Interest Statement

The authors declare that the research was conducted in the absence of any commercial or financial relationships that could be construed as a potential conflict of interest.

## Footnotes

1 http://sciencetechblog.com/2011/05/24/measuring-cell-fluorescence-using-imagej

## Supplementary Material

**FIGURE S1 |** Images depict HPLC chromatograms obtained by injecting either adenosine (ADO) plus inosine (INO) standard solutions (upper panel) or experimental bath samples collected immediately before (Pre-EFS) and after (Post-EFS) stimulation of longitudinal muscle-myenteric plexus (LM-MP) preparations of the ileum from control and TNBS-treated rats. Please note that basal levels of ADO plus INO are inferior in TNBS-treated than in control preparations. Electrical field stimulation (EFS, 5 Hz frequency, 3000 pulses, 1 ms width) increases the ADO plus INO content of bathing samples obtained from both animal groups, but the maximal release of nucleosides was still lower in TNBS-treated rats than in control animals.

## References

[B1] Alves L. A. Bezerra R. J. Faria R. X. Ferreira L. G. da Silva Frutuoso V. (2013). Physiological roles and potential therapeutic applications of the P2X7 receptor in inflammation and pain. *Molecules* 18 10953–10972. 10.3390/molecules180910953 24013409 PMC6270334

[B2] Antonioli L. Fornai M. Colucci R. Awwad O. Ghisu N. Tuccori M. (2011a). Differential recruitment of high affinity A1 and A2A adenosine receptors in the control of colonic neuromuscular function in experimental colitis. *Eur. J. Pharmacol.* 650 639–649. 10.1016/j.ejphar.2010.10.041 21034735

[B3] Antonioli L. Fornai M. Colucci R. Tuccori M. Blandizzi C. (2011b). A holistic view of adenosine in the control of intestinal neuromuscular functions: the enteric ‘purinome’ concept. *Br. J. Pharmacol.* 164 1577–1579. 10.1111/j.1476-5381.2011.01529.x 21658024 PMC3230805

[B4] Arvidsson U. Riedl M. Elde R. Meister B. (1997). Vesicular acetylcholine transporter (VAChT) protein: a novel and unique marker for cholinergic neurons in the central and peripheral nervous systems. *J. Comp. Neurol.* 378 454–467. 10.1002/(SICI)1096-9861(19970224)378:4<454::AID-CNE2>3.0.CO;2-1 9034903

[B5] Barajas-López C. Huizinga J. D. Collins S. M. Gerzanich V. Espinosa-Luna R. Peres A. L. (1996). P2X-purinoceptors of myenteric neurones from the guinea-pig ileum and their unusual pharmacological properties. *Br. J. Pharmacol.* 119 1541–1548. 10.1111/j.1476-5381.1996.tb16070.x 8982499 PMC1915799

[B6] Barros-Barbosa A. R. Ferreirinha F. Oliveira Â. Mendes M. Lobo M. G. Santos A. (2016a). Adenosine A2A receptor and ecto-5′-nucleotidase/CD73 are upregulated in hippocampal astrocytes of human patients with mesial temporal lobe epilepsy (MTLE). *Purinergic Signal.* 12 719–734. 27650530 10.1007/s11302-016-9535-2 PMC5124012

[B7] Barros-Barbosa A. R. Fonseca A. L. Guerra-Gomes S. Ferreirinha F. Santos A. Rangel R. (2016b). Up-regulation of P2X7 receptor-mediated inhibition of GABA uptake by nerve terminals of the human epileptic neocortex. *Epilepsia* 57 99–110. 10.1111/epi.13263 26714441

[B8] Barros-Barbosa A. R. Lobo M. G. Ferreirinha F. Correia-de-Sá P. Cordeiro J. M. (2015). P2X7 receptor activation downmodulates Na^+^-dependent high-affinity GABA and glutamate transport into rat brain cortex synaptosomes. *Neuroscience* 306 74–90. 10.1016/j.neuroscience.2015.08.026 26299340

[B9] Barthó L. Undi S. Benkó R. Wolf M. Lázár Z. Lénárd L. Jr. (2006). Multiple motor effects of ATP and their inhibition by P_2_ purinoceptor antagonist, pyridoxalphosphate-6-azophenyl-2′,4′-disulphonic acid in the small intestine of the guine-pig. *Basic Clin. Pharmacol. Toxicol.* 98 488–495. 10.1111/j.1742-7843.2006.pto_369.x 16635108

[B10] Bodin P. Burnstock G. (2001). Purinergic signalling: ATP release. *Neurochem. Res.* 26 959–969. 10.1023/A:1012388618693 11699948

[B11] Boesmans W. Lasrado R. Vanden Berghe P. Pachnis V. (2015). Heterogeneity and phenotypic plasticity of glial cells in the mammalian enteric nervous system. *Glia* 63 229–241. 10.1002/glia.22746 25161129

[B12] Bogers J. Moreels T. De Man J. Vrolix G. Jacobs W. Pelckmans P. (2000). Schistosoma mansoni infection causing diffuse enteric inflammation and damage of the enteric nervous system in the mouse small intestine. *Neurogastroenterol. Motil.* 12 431–440. 10.1046/j.1365-2982.2000.00219.x 11012943

[B13] Bradley J. S. Jr. Parr E. J. Sharkey K. A. (1997). Effects of inflammation on cell proliferation in the MP of the guinea-pig ileum. *Cell Tissue Res.* 289 455–461. 10.1007/s004410050891 9232824

[B14] Braun N. Sévigny J. Robson S. C. Hammer K. Hanani M. Zimmermann H. (2004). Association of the ecto-ATPase NTPDase2 with glial cells of the peripheral nervous system. *Glia* 45 124–132. 10.1002/glia.10309 14730706

[B15] Broadhead M. J. Bayguinov P. O. Okamoto T. Heredia D. J. Smith T. K. (2012). Ca^2+^ transients in myenteric glial cells during the colonic migrating motor complex in the isolated murine large intestine. *J. Physiol.* 590 335–350. 10.1113/jphysiol.2011.219519 22063626 PMC3285069

[B16] Burnstock G. (1976). Do some nerve cells release more than one transmitter? *Neuroscience* 1 239–248. 11370511 10.1016/0306-4522(76)90054-3

[B17] Bush T. G. Puvanachandra N. Horner C. H. Polito A. Ostenfeld T. Svendsen C. N. (1999). Leukocyte infiltration, neuronal degeneration, and neurite outgrowth after ablation of scar-forming, reactive astrocytes in adult transgenic mice. *Neuron* 23 297–308. 10.1016/S0896-6273(00)80781-3 10399936

[B18] Bush T. G. Savidge T. C. Freeman T. C. Cox H. J. Campbell E. A. Mucke L. (1998). Fulminant jejuno-ileitis following ablation of enteric glia in adult transgenic mice. *Cell* 93 189–201. 10.1016/S0092-8674(00)81571-8 9568712

[B19] Cabarrocas J. Savidge T. C. Liblau R. S. (2003). Role of enteric glial cells in inflammatory bowel disease. *Glia* 41 81–93. 10.1002/glia.10169 12465048

[B20] Carneiro I. Timóteo M. A. Silva I. Vieira C. Baldaia C. Ferreirinha F. (2014). Activation of P2Y6 receptors increases the voiding frequency in anaesthetized rats by releasing ATP from the bladder urothelium. *Br. J. Pharmacol.* 171 3404–3419. 10.1111/bph.12711 24697602 PMC4105929

[B21] Castelucci P. Robbins H. L. Poole D. P. Furness J. B. (2002). The distribution of purine P2X(2) receptors in the guinea-pig enteric nervous system. *Histochem. Cell Biol.* 117 415–422. 10.1007/s00418-002-0404-4 12029488

[B22] Certal M. Vinhas A. Pinheiro A. R. Ferreirinha F. Barros-Barbosa A. R. Silva I. (2015). Calcium signaling and the novel anti-proliferative effect of the UTP-sensitive P2Y11 receptor in rat cardiac myofibroblasts. *Cell Calcium* 58 518–533. 10.1016/j.ceca.2015.08.004 26324417

[B23] Coe I. Zhang Y. McKenzie T. Naydenova Z. (2002). PKC regulation of the human equilibrative nucleoside transporter, hENT1. *FEBS Lett.* 517 201–205. 10.1016/S0014-5793(02)02622-4 12062437

[B24] Collins S. M. Blennerhassett P. Vermillion D. L. Davis K. Langer J. Ernst P. B. (1992a). Impaired acetylcholine release in the inflamed rat intestine is T cell independent. *Am. J. Physiol.* 263(2 Pt 1), G198–G201. 1325127 10.1152/ajpgi.1992.263.2.G198

[B25] Collins S. M. Hurst S. M. Main C. Stanley E. Khan I. Blennerhassett P. (1992b). Effect of inflammation of enteric nerves. Cytokine-induced changes in neurotransmitter content and release. *Ann. N. Y. Acad. Sci.* 664 415–424. 1280933 10.1111/j.1749-6632.1992.tb39780.x

[B26] Correia-de-Sá P. Adães S. Timóteo M. A. Vieira C. Magalhães-Cardoso T. Nascimento C. (2006). Fine-tuning modulation of myenteric motoneurons by endogenous adenosine: on the role of secreted adenosine deaminase. *Auton. Neurosci.* 12 211–224. 10.1016/j.autneu.2006.02.004 16563876

[B27] Costa M. Brookes S. Hennig G. (2000). Anatomy and physiology of the enteric nervous system. *Gut* 47(Suppl. 4), iv15–iv19. 10.1136/gut.47.suppl_4.iv15 PMC1766806 11076898

[B28] Costa M. Brookes S. J. Steele P. A. Gibbins I. Burcher E. Kandiah C. J. (1996). Neurochemical classification of myenteric neurons in the guinea-pig ileum. *Neuroscience* 75 949–967. 10.1016/0306-4522(96)00275-8 8951887

[B29] Davis K. A. Masella J. Blennerhassett M. G. (1998). Acetylcholine metabolism in the inflamed rat intestine. *Exp. Neurol.* 152 251–258. 10.1006/exnr.1998.6839 9710525

[B30] De Man J. G. Seerden T. C. De Winter B. Y. Van Marck E. A. Herman A. G. Pelckmans P. A. (2003). Alteration of the purinergic modulation of enteric neurotransmission in the mouse ileum during chronic intestinal inflammation. *Br. J. Pharmacol.* 139 172–184. 10.1038/sj.bjp.0705218 12746236 PMC1573820

[B31] Diezmos E. F. Sandow S. L. Markus I. Shevy Perera D. Lubowski D. Z. King D. W. (2013). Expression and localization of pannexin-1 hemichannels in human colon in health and disease. *Neurogastroenterol. Motil.* 25 e395–e405. 10.1111/nmo.12130 23594276

[B32] Duarte-Araújo M. Nascimento C. Alexandrina Timóteo M. Magalhães-Cardoso T. Correia-de-Sá P. (2004a). Dual effects of adenosine on acetylcholine release from myenteric motoneurons are mediated by junctional facilitatory A2A and extrajunctional inhibitory A1 receptors. *Br. J. Pharmacol.* 141 925–934. 14993098 10.1038/sj.bjp.0705697 PMC1574269

[B33] Duarte-Araújo M. Nascimento C. Timóteo M. A. Magalhães-Cardoso M. T. Correia-de-Sá P. (2009). Relative contribution of ecto-ATPase and ecto-ATPDase pathways to the biphasic effect of ATP on acetylcholine release from myenteric motoneurons. *Br. J. Pharmacol.* 156 519–533. 10.1111/j.1476-5381.2008.00058.x 19154428 PMC2697673

[B34] Duarte-Araújo M. Timóteo M. A. Correia-de-Sá P. (2004b). Adenosine activating A2A-receptors coupled to adenylate cyclase/cyclic AMP pathway downregulates nicotinic autoreceptor function at the rat myenteric nerve terminals. *Neurochem. Int.* 45 641–651. 10.1016/j.neuint.2004.03.027 15234106

[B35] Dunn K. W. Kamocka M. M. McDonald J. H. (2011). A practical guide to evaluating colocalization in biological microscopy. *Am. J. Physiol. Cell Physiol.* 300 C723–C742. 10.1152/ajpcell.00462.2010 21209361 PMC3074624

[B36] Durnin L. Sanders K. M. Mutafova-Yambolieva V. N. (2013). Differential release of β-NAD^+^ and ATP upon activation of enteric motor neurons in primate and murine colons. *Neurogastroenterol. Motil.* 25 e194–e204. 10.1111/nmo.12069 23279315 PMC3578016

[B37] Eisenman S. T. Gibbons S. J. Verhulst P. J. Cipriani G. Saur D. Farrugia G. (2017). Tumor necrosis factor alpha derived from classically activated “M1” macrophages reduces interstitial cell of Cajal numbers. *Neurogastroenterol. Motil.* 29:e12984. 10.1111/nmo.12984 27781339 PMC5367986

[B38] Ekblad E. Sjuve R. Arner A. Sundler F. (1998). Enteric neuronal plasticity and a reduced number of interstitial cells of Cajal in hypertrophic rat ileum. *Gut* 42 836–844. 10.1136/gut.42.6.836 9691923 PMC1727150

[B39] Engel M. A. Kellermann C. A. Rau T. Burnat G. Hahn E. G. Konturek P. C. (2008). Ulcerative colitis in AKR mice is attenuated by intraperitoneally administered anandamide. *J. Physiol. Pharmacol.* 59 673–689. 19212003

[B40] Epperson A. Hatton W. J. Callaghan B. Doherty P. Walker R. L. Sanders K. M. (2000). Molecular markers expressed in cultured and freshly isolated interstitial cells of Cajal. *Am. J. Physiol. Cell Physiol.* 279 C529–C539. 10913020 10.1152/ajpcell.2000.279.2.C529

[B41] Fields R. D. Burnstock G. (2006). Purinergic signalling in neuron-glia interactions. *Nat. Rev. Neurosci.* 7 423–436. 10.1038/nrn1928 16715052 PMC2062484

[B42] Fujita A. Takeuchi T. Jun H. Hata F. (2003). Localization of Ca^2+^-activated K^+^ channel, SK3, in fibroblast-like cells forming gap junctions with smooth muscle cells in the mouse small intestine. *J. Pharmacol. Sci.* 92 35–42. 10.1254/jphs.92.35 12832853

[B43] Furness J. B. (2006). *The Enteric Nervous System.* Oxford: Blackwell publishing.

[B44] Galligan J. J. (2002). Pharmacology of synaptic transmission in the enteric nervous system. *Curr. Opin. Pharmacol.* 2 623–629. 10.1016/S1471-4892(02)00212-6 12482723

[B45] Giron M. C. Bin A. Brun P. Etteri S. Bolego C. Florio C. (2008). Cyclic AMP in rat ileum: evidence for the presence of an extracellular cyclic AMP-adenosine pathway. *Gastroenterology* 134 1116–1126. 10.1053/j.gastro.2008.01.030 18316082

[B46] Gomez-Pinilla P. J. Gibbons S. J. Bardsley M. R. Lorincz A. Pozo M. J. Pasricha P. J. (2009). Ano1 is a selective marker of interstitial cells of Cajal in the human and mouse gastrointestinal tract. *Am. J. Physiol. Gastrointest. Liver Physiol.* 296 G1370–G1381. 10.1152/ajpgi.00074.2009 19372102 PMC2697941

[B47] Goyal R. K. (2013). Revised role of interstitial cells of Cajal in cholinergic neurotransmission in the gut. *J. Physiol.* 591(Pt 21), 5413–5414. 10.1113/jphysiol.2013.264135 24187080 PMC3936377

[B48] Gulbransen B. D. Bashashati M. Hirota S. A. Gui X. Roberts J. A. MacDonald J. A. (2012). Activation of neuronal P2X7 receptor-Pannexin-1 mediates death of enteric neurons during colitis. *Nat. Med.* 18 600–604. 10.1038/nm.2679 22426419 PMC3321107

[B49] Gulbransen B. D. Sharkey K. A. (2012). Novel functional roles for enteric glia in the gastrointestinal tract. *Nat. Rev. Gastroenterol. Hepatol.* 9 625–632. 10.1038/nrgastro.2012.138 22890111

[B50] Hu H. Z. Gao N. Lin Z. Gao C. Liu S. Ren J. (2002). Chemical coding and electrophysiology of enteric neurons expressing neurofilament 145 in guinea pig gastrointestinal tract. *J. Comp. Neurol.* 442 189–203. 10.1002/cne.1424 11774335

[B51] Hwang S. J. Durnin L. Dwyer L. Rhee P. L. Ward S. M. Koh S. D. (2011). β-nicotinamide adenine dinucleotide is an enteric inhibitory neurotransmitter in human and nonhuman primate colons. *Gastroenterology* 140 608–617. 10.1053/j.gastro.2010.09.039 20875415 PMC3031738

[B52] Jergens A. E. (1999). Inflammatory bowel disease. Current perspectives. *Vet. Clin. North Am. Small Anim. Pract.* 29 501–521. 10.1016/S0195-5616(99)50032-6 10202800

[B53] Kadowaki M. Nagakura Y. Tokita K. Hanaoka K. Tomoi M. (2003). Adenosine A1 receptor blockade reverses experimental postoperative ileus in rat colon. *Eur. J. Pharmacol.* 458 197–200. 10.1016/S0014-2999(02)02766-8 12498926

[B54] Kinoshita K. Horiguchi K. Fujisawa M. Kobirumaki F. Yamato S. Hori M. (2007). Possible involvement of muscularis resident macrophages in impairment of interstitial cells of Cajal and myenteric nerve systems in rat models of TNBS-induced colitis. *Histochem. Cell Biol.* 127 41–53. 10.1007/s00418-006-0223-0 16871386

[B55] Koh S. D. Rhee P. L. (2013). Ionic conductance(s) in response to post-junctional potentials. *J. Neurogastroenterol. Motil.* 19 426–432. 10.5056/jnm.2013.19.4.426 24199003 PMC3816177

[B56] Kukulski F. Lévesque S. A. Sévigny J. (2011). Impact of ectoenzymes on P2 and P1 receptor signaling. *Adv. Pharmacol.* 61 263–299. 10.1016/B978-0-12-385526-8.00009-6 21586362

[B57] Kurashima Y. Amiya T. Nochi T. Fujisawa K. Haraguchi T. Iba H. (2012). Extracellular ATP mediates mast cell-dependent intestinal inflammation through P2X7 purinoceptors. *Nat. Commun.* 3:1034. 10.1038/ncomms2023 22948816 PMC3658010

[B58] Lavoie E. G. Gulbransen B. D. Martín-Satué M. Aliagas E. Sharkey K. A. Sévigny J. (2011). Ectonucleotidases in the digestive system: focus on NTPDase3 localization. *Am. J. Physiol. Gastrointest. Liver Physiol.* 300 G608–G620. 10.1152/ajpgi.00207.2010 21233276

[B59] Lazarowski E. R. Sesma J. I. Seminario-Vidal L. Kreda S. M. (2011). Molecular mechanisms of purine and pyrimidine nucleotide release. *Adv. Pharmacol.* 61 221–261. 10.1016/B978-0-12-385526-8.00008-4 21586361

[B60] Lecci A. Santicioli P. Maggi C. A. (2002). Pharmacology of transmission to gastrointestinal muscle. *Curr. Opin. Pharmacol.* 2 630–641. 10.1016/S1471-4892(02)00225-4 12482724

[B61] Lhermitte F. Gray F. Lyon-Caen O. Pertuiset B. F. Bernard P. (1980). Paralysis of digestive tract with lesions of myenteric plexuses. A new paraneoplastic syndrome. *Rev. Neurol.* 136 825–836. 7291842

[B62] Linden D. R. Couvrette J. M. Ciolino A. McQuoid C. Blaszyk H. Sharkey K. A. (2005). Indiscriminate loss of myenteric neurones in the TNBS-inflamed guinea-pig distal colon. *Neurogastroenterol. Motil.* 17 751–760. 10.1111/j.1365-2982.2005.00703.x 16185315

[B63] MacEachern S. J. Patel B. A. Keenan C. M. Dicay M. Chapman K. McCafferty D. M. (2015). Inhibiting inducible nitric oxide synthase in enteric glia restores electrogenic ion transport in mice with colitis. *Gastroenterology* 149 445.e3–455.e3. 10.1053/j.gastro.2015.04.007 25865048 PMC4516675

[B64] Maes M. Cogliati B. Crespo Yanguas S. Willebrords J. Vinken M. (2015). Roles of connexins and pannexins in digestive homeostasis. *Cell Mol. Life Sci.* 72 2809–2821. 10.1007/s00018-015-1961-8 26084872 PMC4563918

[B65] Marquardt D. L. Gruber H. E. Wasserman S. I. (1984). Adenosine release from stimulated mast cells. *Proc. Natl. Acad. Sci. U.S.A.* 81 6192–6196. 10.1073/pnas.81.19.6192 6435127 PMC391886

[B66] Marques C. C. Castelo-Branco M. T. Pacheco R. G. Buongusto F. do Rosário A. Jr. Schanaider A. (2014). Prophylactic systemic P2X7 receptor blockade prevents experimental colitis. *Biochim. Biophys. Acta* 1842 65–78. 10.1016/j.bbadis.2013.10.012 24184714

[B67] Matteoli G. Gomez-Pinilla P. J. Nemethova A. Di Giovangiulio M. Cailotto C. van Bree S. H. (2014). A distinct vagal anti-inflammatory pathway modulates intestinal muscularis resident macrophages independent of the spleen. *Gut* 63 938–948. 10.1136/gutjnl-2013-304676 23929694

[B68] McGrath J. C. Drummond G. B. McLachlan E. M. Kilkenny C. Wainwright C. L. (2010). Guidelines for reporting experiments involving animals: the ARRIVE guidelines. *Br. J. Pharmacol.* 160 1573–1576. 10.1111/j.1476-5381.2010.00873.x 20649560 PMC2936829

[B69] Mendes C. E. Palombit K. Vieira C. Silva I. Correia-de-Sá P. Castelucci P. (2015). The effect of ischemia and reperfusion on enteric glial cells and contractile activity in the ileum. *Dig. Dis. Sci.* 60 2677–2689. 10.1007/s10620-015-3663-3 25917048

[B70] Miller M. J. Sadowska-Krowicka H. Chotinaruemol S. Kakkis J. L. Clark D. A. (1993). Amelioration of chronic ileitis by nitric oxide synthase inhibition. *J. Pharmacol. Exp. Ther.* 264 11–16. 7678645

[B71] Milusheva E. Sperlágh B. Kiss B. Szporny L. Pásztor E. Papasova M. (1990). Inhibitory effect of hypoxic condition on acetylcholine release is partly due to the effect of adenosine released from the tissue. *Brain Res. Bull.* 24 369–373. 10.1016/0361-9230(90)90091-D 2337817

[B72] Morandini A. C. Savio L. E. Coutinho-Silva R. (2014). The role of P2X7 receptor in infectious inflammatory diseases and the influence of ectonucleotidases. *Biomed. J.* 37 169–177. 10.4103/2319-4170.127803 25116711

[B73] Moreels T. G. De Man J. G. Dick J. M. Nieuwendijk R. J. De Winter B. Y. Lefebvre R. A. (2001). Effect of TNBS-induced morphological changes on pharmacological contractility of the rat ileum. *Eur. J. Pharmacol.* 423 211–222. 10.1016/S0014-2999(01)01088-3 11448487

[B74] Mutafova-Yambolieva V. N. (2012). Neuronal and extraneuronal release of ATP and NAD^+^ in smooth muscle. *IUBMB Life* 64 817–824. 10.1002/iub.1076 22941916 PMC3458179

[B75] Neves A. R. Castelo-Branco M. T. Figliuolo V. R. Bernardazzi C. Buongusto F. Yoshimoto A. (2014). Overexpression of ATP-activated P2X7 receptors in the intestinal mucosa is implicated in the pathogenesis of Crohn’s disease. *Inflamm. Bowel Dis.* 20 444–457. 10.1097/01.MIB.0000441201.10454.06 24412990

[B76] Noronha-Matos J. B. Coimbra J. Sá-e-Sousa A. Rocha R. Marinhas J. Freitas R. (2014). P2X7-induced zeiosis promotes osteogenic differentiation and mineralization of postmenopausal bone marrow-derived mesenchymal stem cells. *FASEB J.* 28 5208–5222. 10.1096/fj.14-257923 25169056

[B77] Nurgali K. Nguyen T. V. Matsuyama H. Thacker M. Robbins H. L. Furness J. B. (2007). Phenotypic changes of morphologically identified guinea-pig myenteric neurons following intestinal inflammation. *J. Physiol.* 583(Pt 2), 593–609. 10.1113/jphysiol.2007.135947 17615102 PMC2277021

[B78] Obata T. Kubota S. Yamanaka Y. (2001). Histamine increases interstitial adenosine concentration via activation of ecto-5′-nucleotidase in rat hearts in vivo. *J. Pharmacol. Exp. Ther.* 298 71–76. 11408527

[B79] Ohta T. Kubota A. Murakami M. Otsuguro K. Ito S. (2005). P2X2 receptors are essential for [Ca^2+^]_i_ increases in response to ATP in cultured rat myenteric neurons. *Am. J. Physiol. Gastrointest. Liver Physiol.* 289 G935–G948. 10.1152/ajpgi.00017.2005 15905416

[B80] Paton W. D. M. Vizi E. S. (1969). The inhibitory action of noradrenaline and adrenaline on acetylcholine output by guinea pig-ileum longitudinal muscle strip. *Br. J. Pharmacol.* 35 10–28. 10.1111/j.1476-5381.1969.tb07964.x 4302725 PMC1703074

[B81] Pelegrin P. Surprenant A. (2006). Pannexin-1 mediates large pore formation and interleukin-1β release by the ATP-gated P2X7 receptor. *EMBO J.* 25 5071–5082. 10.1038/sj.emboj.7601378 17036048 PMC1630421

[B82] Pinheiro A. R. Paramos-de-Carvalho D. Certal M. Costa M. A. Costa C. Magalhães-Cardoso M. T. (2013). Histamine induces ATP release from human subcutaneous fibroblasts, via pannexin-1 hemichannels, leading to Ca^2+^ mobilization and cell proliferation. *J. Biol. Chem.* 288 27571–27583. 10.1074/jbc.M113.460865 23918924 PMC3779754

[B83] Poli E. Lazzaretti M. Grandi D. Pozzoli C. Coruzzi G. (2001). Morphological and functional alterations of the myenteric plexus in rats with TNBS-induced colitis. *Neurochem. Res.* 26 1085–1093. 10.1023/A:1012313424144 11699935

[B84] Pontell L. Castelucci P. Bagyánszki M. Jovic T. Thacker M. Nurgali K. (2009). Structural changes in the epithelium of the small intestine and immune cell infiltration of enteric ganglia following acute mucosal damage and local inflammation. *Virchows Arch.* 455 55–65. 10.1007/s00428-009-0795-x 19517133

[B85] Porcher C. Baldo M. Henry M. Orsoni P. Julé Y. Ward S. M. (2002). Deficiency of interstitial cells of Cajal in the small intestine of patients with Crohn’s disease. *Am. J. Gastroenterol.* 97 118–125. 10.1111/j.1572-0241.2002.05430.x 11808934

[B86] Ren J. Bian X. DeVries M. Schnegelsberg B. Cockayne D. A. Ford A. P. (2003). P2X2 subunits contribute to fast synaptic excitation in myenteric neurons of the mouse small intestine. *J. Physiol.* 552(Pt 3), 809–821. 12937291 10.1113/jphysiol.2003.047944 PMC2343442

[B87] Roberts J. A. Lukewich M. K. Sharkey K. A. Furness J. B. Mawe G. M. Lomax A. E. (2012). The roles of purinergic signaling during gastrointestinal inflammation. *Curr. Opin. Pharmacol.* 12 659–666. 10.1016/j.coph.2012.09.011 23063457 PMC3515696

[B88] Rõszer T. (2015). Understanding the mysterious M2 macrophage through activation markers and effector mechanisms. *Mediators Inflamm.* 2015:816460. 10.1155/2015/816460 26089604 PMC4452191

[B89] Rühl A. Nasser Y. Sharkey K. A. (2004). Enteric glia. *Neurogastroenterol. Motil.* 16(Suppl. 1), 44–49. 10.1111/j.1743-3150.2004.00474.x 15066004

[B90] Sáez P. J. Shoji K. F. Aguirre A. Sáez J. C. (2014). Regulation of hemichannels and gap junction channels by cytokines in antigen-presenting cells. *Mediators Inflamm.* 2014:742734. 10.1155/2014/742734 25301274 PMC4180397

[B91] Sanders K. M. (1996). A case for interstitial cells of Cajal as pacemakers and mediators of neurotransmission in the gastrointestinal tract. *Gastroenterology* 111 492–515. 10.1053/gast.1996.v111.pm8690216 8690216

[B92] Sanovic S. Lamb D. P. Blennerhassett M. G. (1999). Damage to the enteric nervous system in experimental colitis. *Am. J. Pathol.* 155 1051–1057. 10.1016/S0002-9440(10)65207-8 10514387 PMC1867003

[B93] Sawada K. Echigo N. Juge N. Miyaji T. Otsuka M. Omote H. (2008). Identification of a vesicular nucleotide transporter. *Proc. Natl. Acad. Sci. U.S.A.* 105 5683–5686. 10.1073/pnas.0800141105 18375752 PMC2311367

[B94] Sharkey K. A. Kroese A. B. (2001). Consequences of intestinal inflammation on the enteric nervous system: neuronal activation induced by inflammatory mediators. *Anat. Rec.* 262 79–90. 10.1002/1097-0185(20010101)262:1<79::AID-AR1013>3.0.CO;2-K 11146431

[B95] Silva I. Ferreirinha F. Magalhães-Cardoso M. T. Silva-Ramos M. Correia-de-Sá P. (2015). Activation of P2Y6 receptors facilitates nonneuronal adenosine triphosphate and acetylcholine release from urothelium with the lamina propria of men with bladder outlet obstruction. *J. Urol.* 194 1146–1154. 10.1016/j.juro.2015.05.080 26004864

[B96] Sinclair C. J. Shepel P. N. Geiger J. D. Parkinson F. E. (2000). Stimulation of nucleoside efflux and inhibition of adenosine kinase by A1 adenosine receptor activation. *Biochem. Pharmacol.* 59 477–483. 10.1016/S0006-2952(99)00350-0 10660114

[B97] So K. Y. Kim S. H. Sohn H. M. Choi S. J. Parajuli S. P. Choi S. (2009). Carbachol regulates pacemaker activities in cultured interstitial cells of Cajal from the mouse small intestine. *Mol. Cells* 27 525–531. 10.1007/s10059-009-0076-1 19466600

[B98] Stead R. H. Dixon M. F. Bramwell N. H. Riddell R. H. Bienenstock J. (1989). Mast cells are closely apposed to nerves in the human gastrointestinal mucosa. *Gastroenterology* 97 575–585. 10.1016/0016-5085(89)90627-6 2666250

[B99] Stewart T. Beyak M. J. Vanner S. (2003). Ileitis modulates potassium and sodium currents in guinea pig dorsal root ganglia sensory neurons. *J. Physiol.* 552(Pt 3), 797–807. 10.1113/jphysiol.2003.046409 12923214 PMC2343449

[B100] Stoffels B. Hupa K. J. Snoek S. A. van Bree S. Stein K. Schwandt T. (2014). Postoperative ileus involves interleukin-1 receptor signaling in enteric glia. *Gastroenterology* 146 176.e1–187.e1. 10.1053/j.gastro.2013.09.030 24067878

[B101] Takeuchi T. Fujinami K. Goto H. Fujita A. Taketo M. M. Manabe T. (2005). Roles of M2 and M4 muscarinic receptors in regulating acetylcholine release from myenteric neurons of mouse ileum. *J. Neurophysiol.* 93 2841–2848. 10.1152/jn.00986.2004 15574798

[B102] Timóteo M. A. Carneiro I. Silva I. Noronha-Matos J. B. Ferreirinha F. Silva-Ramos M. (2014). ATP released via pannexin-1 hemichannels mediates bladder overactivity triggered by urothelial P2Y6 receptors. *Biochem. Pharmacol.* 87 371–379. 10.1016/j.bcp.2013.11.007 24269631

[B103] Van Assche G. Collins S. M. (1996). Leukemia inhibitory factor mediates cytokine-induced suppression of myenteric neurotransmitter release from rat intestine. *Gastroenterology* 111 674–681. 10.1053/gast.1996.v111.pm8780572 8780572

[B104] Vanderwinden J. M. Timmermans J. P. Schiffmann S. N. (2003). Glial cells, but not interstitial cells, express P2X7, an ionotropic purinergic receptor, in rat gastrointestinal musculature. *Cell Tissue Res.* 312 149–154. 12684872 10.1007/s00441-003-0716-2

[B105] Vasina V. Barbara G. Talamonti L. Stanghellini V. Corinaldesi R. Tonini M. (2006). Enteric neuroplasticity evoked by inflammation. *Auton. Neurosci.* 126–127 264–272. 10.1016/j.autneu.2006.02.025 16624634

[B106] Venkataramana S. Lourenssen S. Miller K. G. Blennerhassett M. G. (2015). Early inflammatory damage to intestinal neurons occurs via inducible nitric oxide synthase. *Neurobiol. Dis.* 75 40–52. 10.1016/j.nbd.2014.12.014 25562655

[B107] Vieira C. Duarte-Araújo M. Adães S. Magalhães-Cardoso T. Correia-de-Sá P. (2009). Muscarinic M3 facilitation of acetylcholine release from rat myenteric neurons depends on adenosine outflow leading to activation of excitatory A_2A_ receptors. *Neurogastroenterol. Motil.* 21 1118–e95. 10.1111/j.1365-2982.2009.01326.x 19470085

[B108] Vieira C. Ferreirinha F. Silva I. Duarte-Araújo M. Correia-de-Sá P. (2011). Localization and function of adenosine receptor subtypes at the longitudinal muscle-myenteric plexus of the rat ileum. *Neurochem. Int.* 59 1043–1055. 10.1016/j.neuint.2011.08.016 21924311

[B109] Vieira C. Magalhães-Cardoso M. T. Ferreirinha F. Silva I. Dias A. S. Pelletier J. (2014). Feed-forward inhibition of CD73 and upregulation of adenosine deaminase contribute to the loss of adenosine neuromodulation in postinflammatory ileitis. *Mediators Inflamm.* 2014:254640. 10.1155/2014/254640 25210228 PMC4152956

[B110] Virginio C. Robertson G. Surprenant A. North R. A. (1998). Trinitrophenyl-substituted nucleotides are potent antagonists selective for P2X1, P2X3, and heteromeric P2X2/3 receptors. *Mol. Pharmacol.* 53 969–973. 9614197

[B111] Vizi E. S. Ono K. Adam-Vizi V. Duncalf D. Földes F. F. (1984). Presynaptic inhibitory effect of Met-enkephalin on [14C] acetylcholine release from the myenteric plexus and its interaction with muscarinic negative feedback inhibition. *J. Pharmacol. Exp. Ther.* 230 493–499. 6747844

[B112] Von Boyen G. Steinkamp M. (2010). The role of enteric glia in gut inflammation. *Neuron Glia Biol.* 6 231–236. 10.1017/S1740925X11000068 21774866

[B113] Wang X. Y. Ward S. M. Gerthoffer W. T. Sanders K. M. (2003). PKC-ε translocation in enteric neurons and interstitial cells of Cajal in response to muscarinic stimulation. *Am. J. Physiol. Gastrointest. Liver Physiol.* 258 G593–G601. 12711590 10.1152/ajpgi.00421.2002

[B114] Ward S. M. Sanders K. M. (2001). Interstitial cells of Cajal: primary targets of enteric motor innervation. *Anat. Rec.* 262 125–135. 10.1002/1097-0185(20010101)262:1<125::AID-AR1017>3.0.CO;2-I 11146435

[B115] Wirtz S. Neurath M. F. (2007). Mouse models of inflammatory bowel disease. *Adv. Drug Rev.* 59 1073–1083. 10.1016/j.addr.2007.07.003 17825455

[B116] Zoppellaro C. Bin A. Brun P. Banzato S. Macchi V. Castagliuolo I. (2013). Adenosine-mediated enteric neuromuscular function is affected during herpes simplex virus type 1 infection of rat enteric nervous system. *PLOS ONE* 8:e72648. 10.1371/journal.pone.0072648 24015268 PMC3754913

