# Supplementary figures and images for: Post-inflammatory Ileitis Induces Non-neuronal Purinergic Signaling Adjustments of Cholinergic Neurotransmission in the Myenteric Plexus
Source: Front Pharmacol. 2017 Nov 8;8:811. doi: 10.3389/fphar.2017.00811 (PMC5682326; doi:10.3389/fphar.2017.00811)

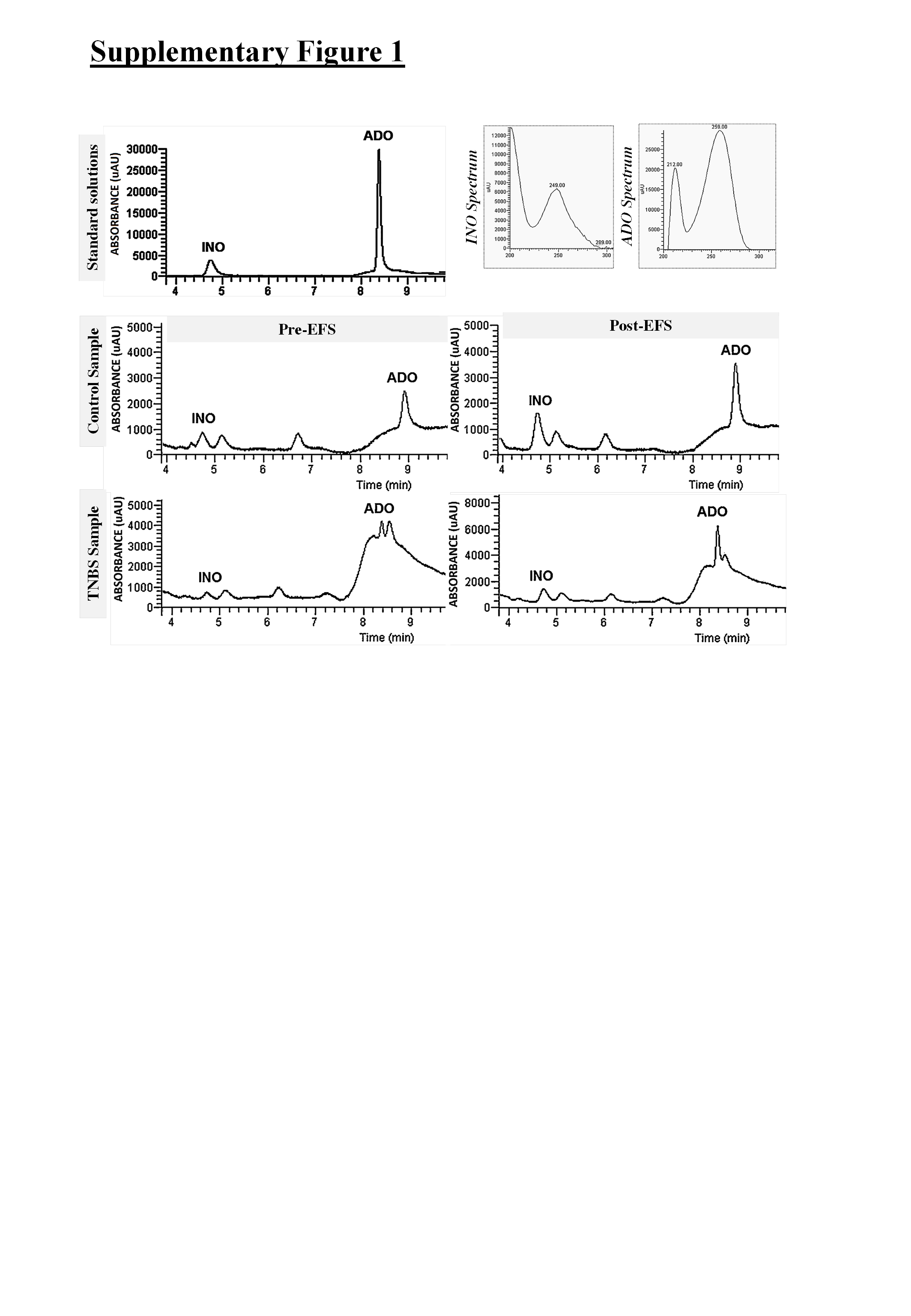

Supplement: Supplementary file 1 [file Image_1.TIF]
